# Increasing airline travel may facilitate co-circulation of multiple dengue virus serotypes in Asia

**DOI:** 10.1371/journal.pntd.0005694

**Published:** 2017-08-03

**Authors:** Huaiyu Tian, Zhe Sun, Nuno Rodrigues Faria, Jing Yang, Bernard Cazelles, Shanqian Huang, Bo Xu, Qiqi Yang, Oliver G. Pybus, Bing Xu

**Affiliations:** 1 State Key Laboratory of Remote Sensing Science, College of Global Change and Earth System Science, Beijing Normal University, Beijing, China; 2 Ministry of Education Key Laboratory for Earth System Modeling, Department of Earth System Science, School of Environment, Tsinghua University, Beijing, China; 3 Department of Zoology, University of Oxford, Oxford, United Kingdom; 4 Ecologie & Evolution, UMR 7625, UPMC-ENS, Paris, France; 5 UMMISCO UMI 209 IRD - UPMC, Bondy, France; Institute for Disease Modeling, UNITED STATES

## Abstract

The incidence of dengue has grown dramatically in recent decades worldwide, especially in Southeast Asia and the Americas with substantial transmission in 2014–2015. Yet the mechanisms underlying the spatio-temporal circulation of dengue virus (DENV) serotypes at large geographical scales remain elusive. Here we investigate the co-circulation in Asia of DENV serotypes 1–3 from 1956 to 2015, using a statistical framework that jointly estimates migration history and quantifies potential predictors of viral spatial diffusion, including socio-economic, air transportation and maritime mobility data. We find that the spread of DENV-1, -2 and -3 lineages in Asia is significantly associated with air traffic. Our analyses suggest the network centrality of air traffic hubs such as Thailand and India contribute to seeding dengue epidemics, whilst China, Cambodia, Indonesia, and Singapore may establish viral diffusion links with multiple countries in Asia. Phylogeographic reconstructions help to explain how growing air transportation networks could influence the dynamics of DENV circulation.

## Author summary

In the past half century the incidence of dengue fever worldwide has increased 30-fold, with an estimated ~390 million infections per year. The years 2014 and 2015 were characterized by large dengue outbreaks worldwide, which are a threat to public health, especially in Asian countries. Here we use a phylogeographic approach to reconstruct historical virus movement and evaluate multiple potential predictors of the spatial spread of dengue virus (DENV) among Asian countries. We show that air traffic has played a more important role than maritime transport in shaping large-scale geographic dispersal of DENV in Asia. Our study suggests that the patterns of DENV spread result from the interplay between intensity and structure of human mobility through the air transportation network.

## Introduction

Dengue virus (DENV) is a growing threat to public health, with nearly 390 million infections every year worldwide, of which ~96 million are symptomatic [1,2]. An estimated 2.5 billion people are at risk of dengue infection [3]. Dengue is regarded as the world’s most important mosquito-borne viral disease and is endemic in more than 100 countries [4], with most disease burden limited to tropical and subtropical regions [5]. However, in 2014 an outbreak of dengue occurred in Japan for the first time in over 70 years. This occurred despite the country’s temperate climate, and viral phylogenetic analysis suggests that the Japanese outbreak resulted from international travel from Southeast Asia [6]. Furthermore, the number of reported dengue cases and outbreaks continues to increase. In 2014, a dengue outbreak affected several Asian countries, including China, Thailand, Vietnam and Japan [7,8].

The majority of DENV infections are asymptomatic or cause a mild febrile disease known as dengue fever, while the more severe forms of dengue infection—dengue hemorrhagic fever (DHF) and dengue shock syndrome (DSS)—may be life threating with >20% mortality [9]. Epidemiological studies have indicated that DHF often occurs when a dengue-immune person acquires a second infection with a different DENV serotype [10,11], and it has been hypothesized that DHF/DSS may result from a process of antibody-dependent enhancement [12,13]. The geographical areas in which transmission of multiple dengue serotypes occurs has grown in recent years and the pattern of co-circulation is conspicuously different from that which prevailed decades ago [14,15]. Furthermore, a growing number of DHF/DSS cases in the last 50 years, especially in Asia, demonstrates the need for a better understanding of how DENV genetic diversity and transmission jointly shape dengue epidemics.

The growing scale of human mobility, particularly through air transportation, underscores an increase of pathogen introductions into geographic areas suitable for transmission, potentially contributing to the emergence and the re-emergence of epidemics [16,17]. Recent examples include the SARS epidemic, novel influenza A virus strains, the Middle East respiratory syndrome (MERS) coronavirus, and Zika virus [18–21]. At local scales, endemic circulation of DENV strains can be driven by viral lineage introduction events that eventually lead to lineage replacement [22] and viral introductions are expected to increase with human mobility. Together, these observations suggest that increasing human mobility through air-traffic networks may impact the spread of pathogens at large geographical scales. Here we combine Asian socio-economic data, air and maritime transportation network data and viral genetic data to identify key drivers of the spread of DENV serotypes 1, 2 and 3 in Asia, and to reconstruct the patterns of virus circulation among Asian countries over the past half century.

## Materials and methods

### Compilation of genetic datasets

**Fig 1:**
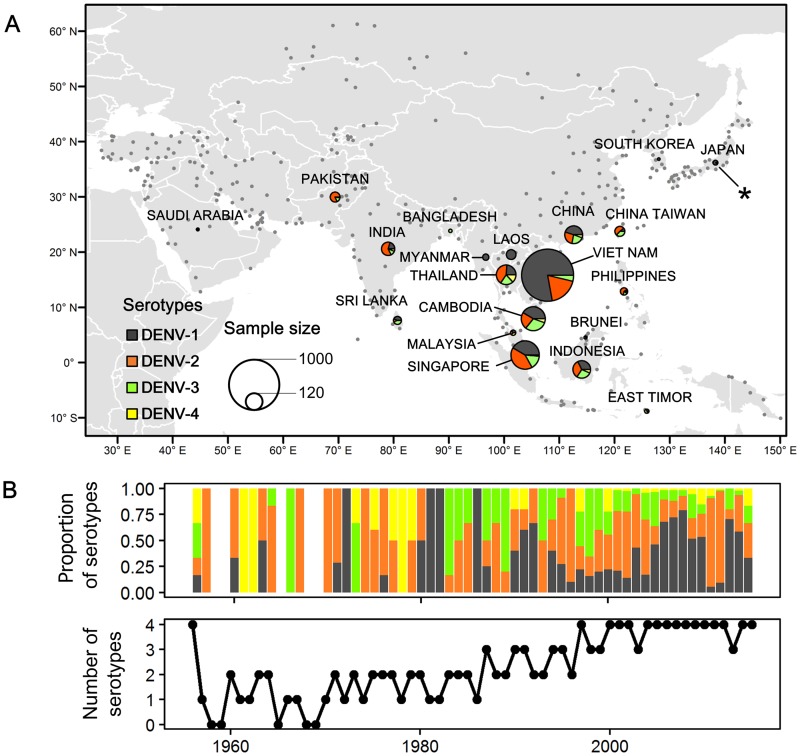
Co-circulation of dengue virus serotypes in Asia. (A). Locations of available viral sequences (within regions defined by sampling effort) and airports. Circle areas are proportional to the number of genetic sequences from a particular geographic location and for a given serotype (dark grey: DENV-1, orange: DENV-2, green: DENV-3, and yellow: DENV-4). Grey dots represent the airports for which passenger flux data was used in the analysis. Sequences obtained from patients in Japan, 2014 are indicated by asterisk. (B) The upper panel shows the proportion of sequences from each serotype per year, while the lower panel shows the number of serotypes isolated per year in Asia. Co-occurrence of multiple serotypes in a single year has become increasingly frequent.

Maximum phylogenetic information would be obtained by studying whole DENV genomes. However, available DENV whole genomes from Asia have insufficient coverage through time and space for reliable analysis, and most available sequences comprise partial or complete coding E gene sequences. In order to generate a data set with both acceptable phylogenetic diversity and spatiotemporal sampling, we used DENV E gene sequences in subsequent analyses. DENV (DENV-1 to DENV-3) envelope (E) gene sequences with known collection dates and locations of sampling in Asia were collected from GenBank. DENV-4 was not included in this study because too few samples were available (only 64 sequences from 11 countries; Fig 1). The remaining strains comprised a total of 2,202 sequences sampled between 1956 and 2015, from 20 distinct countries or geographic regions (Fig 1). Sequences were grouped by serotype and aligned separately using MAFFT [23]. Recombination was inspected using the methods implemented in RDP3 and SimPlot [24]. After removing duplicate and recombinant strains, the final data set contained 1,272 DENV-1 sequences, 628 DENV-2 sequences and 302 DENV-3 sequences. In the complete sequence dataset, some countries, such as Vietnam, Cambodia, Thailand, and Singapore, were over-represented. In order to control for possible bias from uneven sampling, we randomly subsampled the complete sequence datasets by location and sampling time. At most 10 sequences were sampled per country and per year in order to create a more equitable spatio-temporal sampling distribution. After sub-sampling, the total number of sequences analyzed here was 327 for DENV-1, 357 for DENV-2, and 202 for DENV-3, sampled over a total of 59 years (S1 Fig). Details of the sequences in each data set, including information on the year of isolation, sampling location, and accession numbers, are provided in Supplementary Information (S1 Table).

### Time-scaled phylogenetic tree reconstruction

For each serotype subsampled data set, we first estimated the correlation between root-to-tip genetic divergence and sequence sampling dates, using TempEst [25]. This preliminary analysis indicated a good temporal signal for all serotypes (S2 Fig). To reconstruct past population dynamics, we used a coalescent-based Gaussian Markov random field (GMRF) method with the time-aware smoothing parameter [26], as implemented in BEAST v1.8.2 [27]. A GTR+I+Γ nucleotide substitution model and an uncorrelated lognormal relaxed molecular clock model were used, with a prior distribution for the evolutionary rate parameter set to a Γ distribution with shape = 0.001 and scale = 1000. The BEAGLE library was used to accelerate computation [28]. For each serotype, three independent analyses of 150 million generations were performed, sampling parameters and trees every 15,000 generations. Analyses were combined after the removal of a burn-in of 10–20% of the samples and were checked visually in Tracer v.1.5.

### Estimating viral migration through discrete geographic locations

To reconstruct the spatial dynamics of DENV-1–3, we used a Bayesian Markov chain Monte Carlo (MCMC) phylogeographic discrete approach [29,30] that estimates the ancestral locations along each branch of a viral phylogeny, as implemented in BEAST v1.8.2 [27]. A GTR+ I+Γ nucleotide substitution model was used in this analysis. Three independent MCMC chains were run for 150 million states, sampling every 15,000 states after the removal of 10% burn-in. Maximum clade credibility (MCC) trees were summarized using TreeAnnotator and visualized using SPREAD [31].

To provide a minimal set of location state changes that provide an adequate description of viral spread, we used Bayesian stochastic search variable selection (BSSVS) [30,32]. BSSVS uses Bayes factors (BF) and binary indicator variables (I) to identify statistically supported viral lineage movement routes. Values of BF > 6 and I > 0.5 were considered as denoting a significant migration pathway, where BF > 1,000 indicates decisive statistical support, 100 ≤ BF < 1,000 indicates very strong support, 30 ≤ BF < 100 indicates strong support, 10 ≤ BF < 30 indicates substantial support and 6 ≤ BF < 10 indicates support [32]. We next estimated the number of expected transitions among location-pairs (“Markov jum” counts) along the phylogeny branches [33], which provided a quantitative measure of successful viral introductions among countries [34].

### Air transportation network in Asia

We investigated the air transportation network in Asia using mobility data from ICAO (International Civil Aviation Organization; http://www.icao.int/Pages/default.aspx). This database contains the number of passengers traveling among 373 airports in Asia during the years 1982, and 1992–2012, and included all scheduled flights both for large and small aircraft. Country-level movement of passengers was obtained by aggregating airport-level movement for each country.

To obtain insight into temporal changes across the air transportation network in Asia, directed and weighted air flow networks were constructed, with countries as the network nodes. Network edges were weighted by the number of air passengers connecting pairs of countries. Hubs within the Asian air transportation network were also identified using two network topology properties: degree centrality and betweenness centrality. Degree centrality measures the number of edges connected to a node. Here, the degree of a given country refers to the number of airlines linking to it in the airline network. Betweenness centrality depends on the proportion of shortest paths between all pairs of vertices that pass through a given node. Thus the betweenness centrality of a given country is a measure of the extent to which a country lies on routes between other countries in the airline network and is calculated as:

$$Betweenness\;of\;node\;k=\sum_{s\neq v\neq t}\frac{\sigma_{st}\left(k\right)}{\sigma_{st}}$$ (1)

where *σ*_st_ is the total number of shortest paths from node *s* to node *t* and *σ*_st_ (k) is the number of those paths that pass through *k*.

### Maritime transportation network in Asia

To represent the shipping connectivity between pairs of countries, the liner shipping bilateral connectivity index (LSBCI) in Asia from 2008 to 2014 was obtained from United Nations Conference on Trade and Development (UNCTAD; http://unctadstat.unctad.org/EN/Index.html), which captures the amount of goods transported between two countries. Container port throughput (CPT) for each Asian country was also obtained from UNCTAD.

### Identifying potential determinants of DENV lineage movement

To investigate the potential predictors driving DENV-1, DENV-2 and DENV-3 spatial spread, we used a generalized linear model (GLM) extension to Bayesian phylogeographic inference [20,35]. The phylogeographic GLM method parameterizes the continuous-time Markov chain (CTMC) matrix of among-location lineage migration parameters as a log-linear function of several potential predictors. Each function includes a coefficient *β* (in log space) and a binary indicator variable *δ*. BSSVS [30] was used to estimate the posterior probability that each predictor is included or excluded from the model. We considered several potential predictors of DENV diffusion. These included log-transformed and standardized measures of demographic and economic data, geographical distances among countries, absolute latitude, DENV sample sizes. Finally, predictors of human mobility from shipping connectivity and air transportation movement were included (averaged values were estimated using data in 1982, 2000, and 2012).

Specific details of potential predictors are as follows: (i) average distance between two countries was estimated as the average of pairwise distances between all pairs of airports in the two countries, (ii) absolute latitudes for each country were calculated as the latitudes of their geometric center, (iii) passenger flow represents the number of passengers on fights between each pair of countries, (iv) LSBCI (linear shipping bilateral connectivity index) represents goods transported by the sea between pairs of countries, (v) population sizes and densities for each country in 2000 were obtained from the UN World Population Prospects database (http://www.un.org/en/development/desa/population/), (vi) gross domestic product (GDP) for each country in 2000 was collected from the UN Statistics Division (https://unstats.un.org) and (vii) average temperature and rainfall for each Asian country was extracted from the data set provided by WorldClim [36]. In addition, we included sample sizes (number of dengue sequences per country) for origin and destination locations as potential predictor variables, in order to test the impact of heterogeneous sampling.

## Results

### Evolutionary history of dengue virus serotypes in Asia

Fig 1A depicts the geographic locations of the sequences used in this study, from a total of 20 distinct countries or geographic regions in Asia; most sequences were sampled in Southeast Asia. To reduce potential bias in the reconstruction of spatial spread that may arise from over-sampling particular locations, we subsampled the original data (see Methods). It is important to note that, in recent years, particularly since the 1980s, genetic evidence of multiple dengue virus sequences from a given location has increased (Fig 1B) in Asian countries (S3 Fig).

Evolutionary analysis of 327 DENV-1 E gene sequences showed that Asian sequences fell into five distinct lineages, genotypes I—V. The 357 DENV-2 E gene sequences were classified into five genotypes: Asia I, Asia II, America/Asia, Cosmopolitan, and the America genotype. Further, the 202 DENV-3 E gene sequences comprised four genotypes, genotypes I and III—V. The estimated evolutionary rates of the E gene of each serotype, under the selected evolutionary model, were 7.50×10^−4^ (95% highest posterior density, HPD: 6.73×10−4–8.28×10^−4^) substitutions per site per year (s/s/y) for DENV-1, 8.14×10^−4^ (95% HPD: 7.38×10−4–8.94×10^−4^) s/s/y for DENV-2, and 7.96×10^−4^ (95% HPD: 7.01×10−4–8.89×10^−4^) s/s/y for DENV-3. The corresponding estimates of the time of the most recent common ancestors (TMRCAs) of DENV-1–3 in Asia were 1911 (95% HPD: 1891–1928), 1901 (95%HPD 1868–1931, after removal of sylvatic lineages) and 1929 (95% HPD: 1916–1941) respectively.

### Asian airline network growth

**Fig 2:**
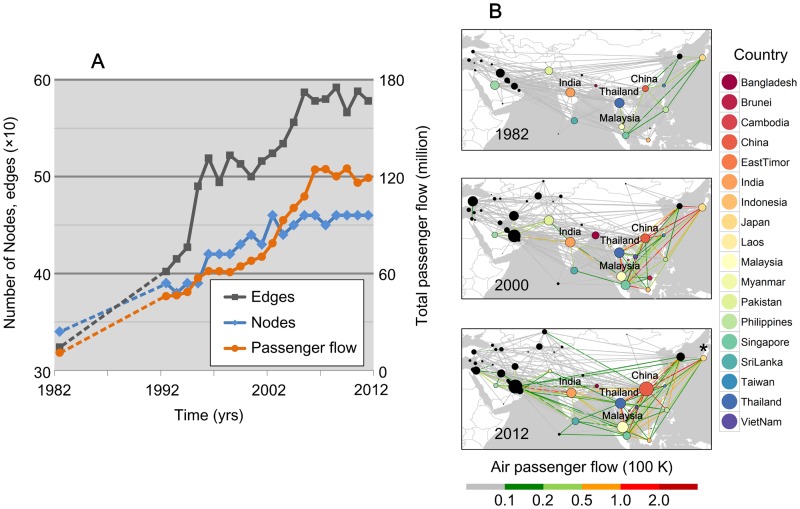
Growing airline networks in Asia. (A) Number of nodes (countries belonging to the Asian air transport network), the number of airlines, and total passenger flow in the Asian air transport network between 1982–2012. Records are not available for the period 1983–1991, so dashed lines represented imputed missing values obtained by linear interpolation. (B) Passenger flux in Asia in 1982, 2000 and 2012. The size of each node corresponds to the degree centrality of the country in the airline network. Color coded lines represent the volume of air passenger flux. Node colors correspond to the countries indicated on the right-hand side of the figure. Black points indicate countries for which no virus sequence was included in the genetic analyses. China shares one of the busiest Asian flight routes with Japan, which is indicated by an asterisk.

Air transportation has historically exhibited significant growth [37]. We found that Asian air transportation grew substantially from 1980 onwards (Fig 2A). We observe that several countries act as hubs in airline network, e.g. India, Singapore, and Thailand, while other countries exhibit growing network centrality over time, such as China and Malaysia (Fig 2B). It is interesting to note that the sequences obtained from dengue patients in 2014 in Japan shared a very high identity with a sequence from China, which shares one of the busiest Asian flight routes with Japan (Fig 2B). These observations prompt us to question the role of air passenger transportation in DENV spread in Asia.

### Air travel may drive the spatial spread of DENV in Asia

**Fig 3:**
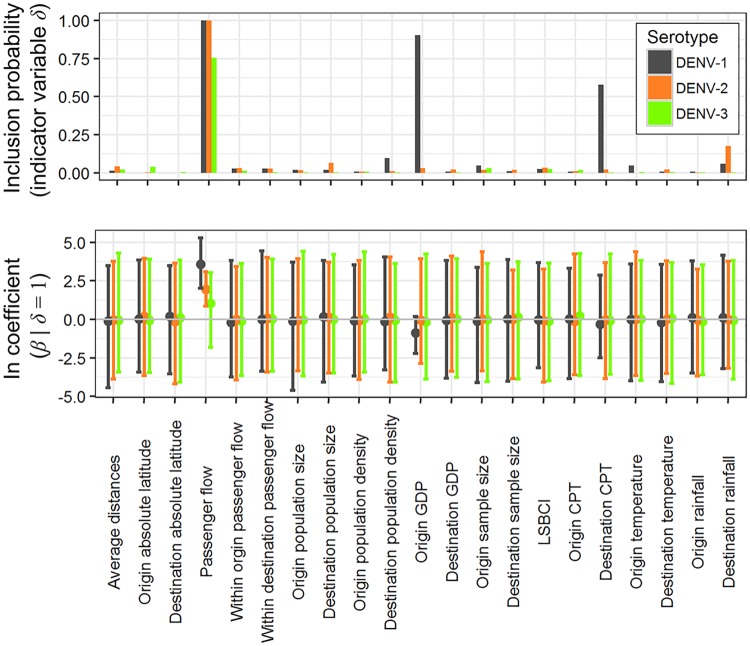
Predictors of DENV-1–3 diffusion in Asia. In the top panel bars show *δ*, an indicator variable that governs the probability of inclusion or exclusion of the predictor in the model. In the lower panel, points and error bars indicate the mean and the 95% Bayesian credible intervals, respectively, of the estimated conditional effect size of the GLM coefficients (*β*|*δ* = 1) on a log scale, for each predictor variable. *β* is the effective size of the predictor variable. GDP: Gross domestic product; LSBCI: linear shipping bilateral connectivity index; CPT: container port throughput.

To infer the contribution of candidate factors driving DENV diffusion in Asia we used a generalized linear model that simultaneously estimates ancestral geographic reconstruction and identifies the contribution of potential predictors of spatial spread [20,35]. Our results show that most candidate predictors of virus spread, such as geographic distance and demographic factors, are not significantly associated with viral spread (Fig 3). However, we do find that air passenger flow is a dominant driver of DENV lineage movement and the inclusion of this factor in the model is supported for all three DENV serotypes. GDP at the location of origin and CPT (container port throughput) at the destination location are negatively associated with DENV-1 lineage movement in Asia, but not for the other two genotypes (Fig 3). Sample sizes for each location are not associated with viral lineage movement, which suggests that our conclusions are not driven by sampling biases.

### Viral migration through discrete geographic locations

**Fig 4:**
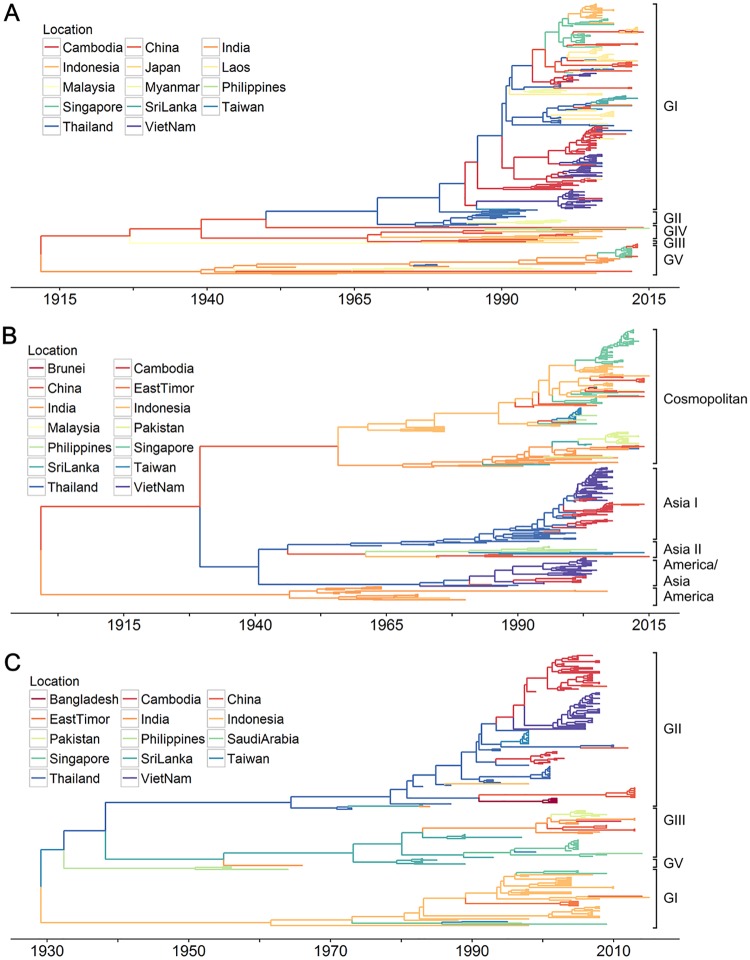
Maximum clade credibility trees of E gene of dengue virus serotypes in Asia. (A) Phylogeographic molecular clock phylogeny of DENV-1 (n = 327), (B) DENV-2 (n = 357), (C) DENV-3 (n = 202, after removal of sylvatic lineages). Branches are colored according to most probable geographic location, as inferred using a Bayesian discrete non-reversible phylogeographic approach.

**Fig 5:**
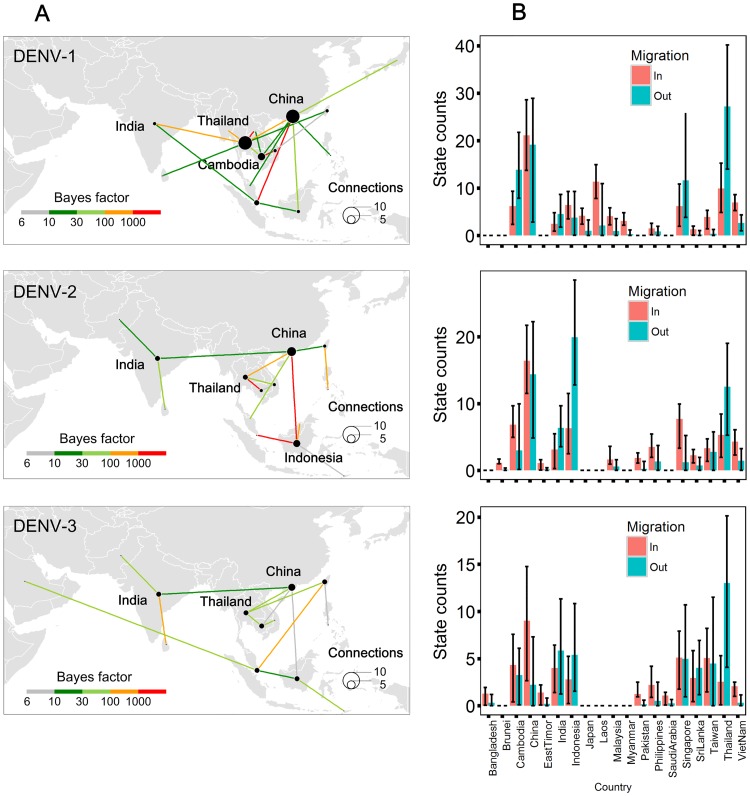
Strongly supported pathways of lineage movement and histograms of the total number of location state transitions of DENV-1–3 in Asia for 1956 to 2015. (A) Strongly supported state transitions, indicating migration of DENV-1–3 lineages among discrete locations. The size of points corresponds to the number of geographic connections. The colored lines represent statistical support for a given viral movement pathway. Only those viral lineage movements supported with a BF > 6 are shown. (B) Number of expected transitions into and out of each country per serotype. Error bars represent 95% BCIs.

To understand the spatial circulation of DENV in Asia, we reconstructed the past spatial transmission patterns of serotypes DENV-1–3 over the study period, for each country from which sequences were sampled (Fig 4). A Bayesian stochastic search variable selection procedure was employed to infer a minimum set of location exchange events, while a Markov jump (MJ) analysis was used to quantity viral lineage movement among pairs of locations (see Methods). The phylogeographic analysis in Fig 5 indicates several significant migration links (with BF support > 1000). For DENV-1 these are between Cambodia and Vietnam, Thailand and Laos, and Singapore and China. For DENV-2, the well supported links are between Thailand and Cambodia, China and Indonesia, and Indonesia and Singapore. Finally, for DENV-3 the strongly supported links are between India and Sri Lanka (BF > 800). We also inferred a number of other well supported movements among countries; a full list is provided in S2 Table.

**Fig 6:**
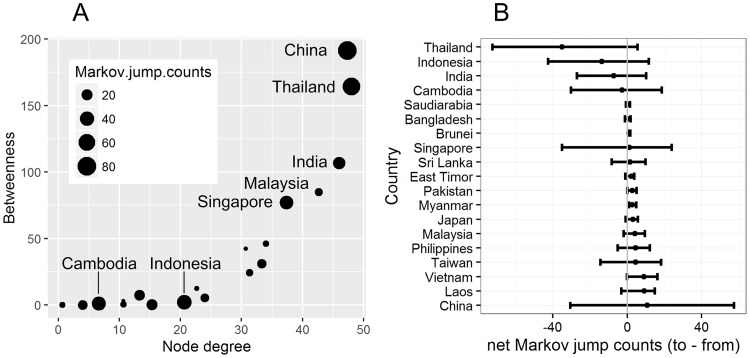
Centrality measures of the air passenger network and number of state transitions. (A) Scatterplot of centrality measures for the air travel network based on degree and betweenness. The “degree” centrality of a given country (node) refers to the number of airlines linking it in the airline network, and the “betweenness” centrality of a given country measures the extent to which a country lies on routes between other countries in airline network (see Methods). The size of each point corresponds to the number of expected Markov jump transitions (including both importations and exportations). (B) Net Markov jump counts, summed across all 3 DENV serotypes. For each country, we summarize the average net Markov jumps (jumps to—jumps from) and their 95% credible intervals. The estimates are ordered from the lowest to highest number of net jumps.

We further find that estimated number of viral lineage migrations (including both importations and exportations) for each country is associated with measures of centrality in the air transportation network (Pearson correlation: r = 0.45, *P* = 0.05, for degree centrality and state transitions; and r = 0.73, *P* < 0.01, for betweenness centrality and state transitions; Fig 6A, S4 Fig). However, countries do not all contribute equally to viral lineage dissemination. An analysis of the number of virus lineage movements and the air transportation network analysis for each country indicates increased lineage virus movements from Thailand, India, and Indonesia to other locations [38,39], and a trend towards viral lineage importation from other locations for Vietnam and China. Lineage import and export is approximately equal for Cambodia and Singapore (Fig 6B and S5 Fig). Given the betweenness centrality of Thailand and India in the regional airline network, these countries may act as net sources of viral lineages, while China, Cambodia, Indonesia and Singapore may establish strong links with multiple countries within the network.

There has been an increasing frequency of DENV lineage co-occurrence in China over time, concomitant with its increasing centrality in the Asian air transportation network (Figs 2 & 5). In contrast, Vietnam, a dengue-endemic country, has a low frequency of viral lineage import and export with other locations (Fig 6B), likely due to its lower network centrality. However, we find that some non-endemic countries that do not contribute significantly to viral lineage movement across Asia may still maintain high airline passenger flows, such as Japan, South Korea, and countries in Western Asia. This may be because such areas lack a climate suitable for widespread dengue virus transmission. Further we are not able to infer transmission between countries if no viral sequences are available for those locations. The results here do not appear to be driven by heterogeneous sampling; similar results were obtained using downsampled datasets with a maximum of 5 sequences per country per year (S6 Fig).

## Discussion

Our study provides a temporal description of the patterns of DENV spread across Asia. The spatial dynamics of dengue virus in Asia inferred here suggests that DENV genetic diversity in Asia is dynamic yet spatially structured, with frequent virus lineage movement among countries, and frequent co-circulation of dengue virus lineages. Our analysis suggests that the air transportation network has contributed to the spatial distribution of DENV serotypes in Asia. This can be explained by the movement of viraemic individuals; if the destination and timing of virus introductions coincides with a climatically suitable period then an epidemic can become established in a susceptible recipient population. Further, it is possible that the mobility of viraemic mosquitoes through air transportation networks is non-negligible [40]. For example, one study estimated that 8–20 *Anopheline* mosquitoes were imported into France per flight in one 3-week period in 1994 [41]. Previous studies have also shown that mosquito species, including *Aedes albopictus*, can survive long-haul flights [42,43]. Implementing disinfection is proving effective in vector control and disease prevention [40,44], highlighting the importance of air transportation in pathogen importation.

The positive coefficient *β* for the air traffic predictor in our phylogenetic GLM model indicates that DENV lineage dispersal is associated with air traffic; this result is consistent with previous studies of DENV in Brazil, which concluded that air travel of humans and/or mosquitoes is associated with virus lineage movement [22]. Notably there is strong statistical support for the inclusion of the air travel predictor variable in the GLM models for all three DENV serotypes in our study. Further, we find that origin GDP and destination CPT (container port throughput) are negatively related to DENV diffusion for DENV-1; this result is possibly linked to better public health infrastructure and vector control programmes in wealthier countries [45]. However, there was no support for the inclusion of those two predictors in the analyses of DENV-2–3. This may stem from the larger number of sequences for DENV-1, or from differences in sampling distribution among countries, despite the fact we have used a subsampling procedure in an attempt to mitigate potential bias. Alternatively, observed among-genotype differences may be driven solely by stochastic variation in the process of spatial dissemination; for example, the well-supported migration links inferred using DENV-1–3 sequences were not identical (Fig 5).

Analysis of the air transportation network indicates differences in the roles of air transport nodes in the regional-scale dissemination of DENV lineages. Our data suggest that large transportation hubs account for most of the inferred virus lineage movements within their respective networks. For example, Thailand and India seem to act as central hubs of DENV lineage movement in Asia, demonstrating both inward/outward viral migrations with other countries in the region. In contrast, our results suggests that the low level of network centrality of Vietnam may explain its limited role in regional DENV geographical dissemination. Some countries, such as China and Malaysia, may have had an increasing role in DENV spread through time. We note that dengue is still considered to be an imported disease in mainland China [46]. It might be expected that some DENV endemic countries, with climatic suitability for dengue transmission and high betweenness-centrality in air transportation network, contribute to seeding Asian dengue epidemics. However, it has been shown that the risk factors for the persistence and transmission of dengue are complex [47], once the socioeconomic conditions were taken into account (as shown in Fig 3).

Previous studies based on simulation models have shown that global warming is likely to increase the area of land with a climate suitable for dengue virus transmission by altering the distribution of *Aedes aegypti*, the main mosquito vector of dengue [48,49]. This may result in more countries being at risk of dengue virus via the air transportation network, resulting in the potential for spread across even greater geographic scales. Temperature increases may lead to lengthened mosquito lifespans and shortened extrinsic incubation periods, which may result in more infected mosquitoes for a longer period of time [50,51].

Our study has several limitations. First, we cannot discern the relative contribution of infected humans versus infected mosquitoes in the spread of DENV, which requires more detailed epidemiological data and pathogen genome sequences from both mosquitoes and patients in future studies. However the information provided by the genetic analyses here can be useful to parameterize future spatially-structured transmission models. For example, the structure and travel flux of the airline transport network, as well as the among-country rates of lineage movement from the phylogeographic model, could be used to inform future simulations [20]. Second, our findings are based on DENV E genes for Asian countries, as this is the only genome region for which sufficient numbers of sequences are available, yet complete virus genomes would provide better phylogenetic resolution [52,53]. Further, our analyses were limited to DENV in Asia and do not consider transmission to or from other regions. Finally, the uneven sampling of DENV sequences in Asia, especially in a few countries after 2000 (e.g. Vietnam and Singapore) necessitated the use of sub-sampling analyses that accounted for the number of samples per location. Results obtained from a more representative data set indicates that our main conclusions are robust to sample sizes.

Future trends in global mobility could potentially accelerate the appearance and diffusion of DENV worldwide. Prevention and control of dengue epidemics requires a better understanding of its mode of geographical dissemination, especially for countries in the tropics. Our study highlights the importance of developing a multivalent DENV vaccine in order to cope with increasing frequency of DENV serotype co-occurrence. The potential impacts of vaccination on dengue epidemics in Asia should be considered [54]. Viral spatial dissemination, together with host age-structure and host-vector interactions are required in mathematical models to inform future vaccine deployment decisions.

## Supporting information

S1 Fig Raw data and subsampled data of DENV sequences in each country in each year in Asia. The color spectrum shows the number of sequences from each year and location, from grey (low numbers of sequences) to dark red (high numbers of sequences). The raw data set (left column) contained 1272 DENV-1 sequences, 628 DENV-2 sequences and 302 DENV-3 sequences. After randomly subsampling by location and sampling time (right column) the total number of subsampled sequences analyzed were 327 for DENV-1, 357 for DENV-2, and 202 for DENV-3.
(TIF)

S2 Fig Plots of the root-to-tip genetic distance against sampling time for different serotype subsample data sets (DENV-1, DENV-2, and DENV-3). TempEst was used to obtain exploratory regressions based on the maximum likelihood trees.
(TIF)

S3 Fig Number of serotypes isolated in each country in each year for the raw data set (i.e. before subsampling). (TIF)

S4 Fig Scatter plot of “Markov jump” counts, betweenness and degree of air flow network for each country. (TIFF)

S5 Fig Net Markov jump counts across for each DENV serotype. For each country, we summarize the average net Markov jumps (jumps to—jumps from) and their 95% credible intervals. The estimates are reordered from the lowest to highest net jumps.
(TIF)

S6 Fig Histograms of total number of state transitions in each country for each serotype under different subsampling methods. Error bars represent 95% highest posterior density intervals. Left panel: at most 10 sequences were sampled per country per year. Middle panel: at most 5 sequences were sampled per country per year. Right panel: scatter plot of state transitions of the two subsampled data sets.
(TIF)

S1 Table Subsampled DENV E gene sequences in study. (DOCX)

S2 Table Strongly supported DENV migrations estimated from E gene segment. (DOCX)

## References

[1] Bhatt S, Gething PW, Brady OJ, Messina JP, Farlow AW, et al (2013) The global distribution and burden of dengue. Nature 496: 504–507. doi: 10.1038/nature12060 23563266 10.1038/nature12060 PMC3651993

[2] Brady OJ, Gething PW, Bhatt S, Messina JP, Brownstein JS, et al (2012) Refining the global spatial limits of dengue virus transmission by evidence-based consensus. PLoS Negl Trop Dis 6: e1760 doi: 10.1371/journal.pntd.0001760 22880140 10.1371/journal.pntd.0001760 PMC3413714

[3] Luz PM, Vanni T, Medlock J, Paltiel AD, Galvani AP (2011) Dengue vector control strategies in an urban setting: an economic modelling assessment. Lancet 377: 1673–1680. doi: 10.1016/S0140-6736(11)60246-8 21546076 10.1016/S0140-6736(11)60246-8 PMC3409589

[4] Guzman MG, Harris E (2015) Dengue. Lancet 385: 453–465. doi: 10.1016/S0140-6736(14)60572-9 25230594 10.1016/S0140-6736(14)60572-9

[5] Raghwani J, Rambaut A, Holmes EC, Hang VT, Hien TT, et al (2011) Endemic dengue associated with the co-circulation of multiple viral lineages and localized density-dependent transmission. PLoS Pathog 7: e1002064 doi: 10.1371/journal.ppat.1002064 21655108 10.1371/journal.ppat.1002064 PMC3107208

[6] Quam MB, Sessions O, Kamaraj US, Rocklöv J, Wilder-Smith A (2016) Dissecting Japan's dengue outbreak in 2014. Am J Trop Med Hyg 94: 409–412. doi: 10.4269/ajtmh.15-0468 26711518 10.4269/ajtmh.15-0468 PMC4751952

[7] Tian H, Huang S, Zhou S, Bi P, Yang Z, et al (2016) Surface water areas significantly impacted 2014 dengue outbreaks in Guangzhou, China. Environ Res 150: 299–305. doi: 10.1016/j.envres.2016.05.039 27336234 10.1016/j.envres.2016.05.039

[8] Wang S, Wang W, Chang K, Chen Y, Tseng S, et al (2016) Severe dengue fever outbreak in Taiwan. Am J Trop Med Hyg 94: 193–197. doi: 10.4269/ajtmh.15-0422 26572871 10.4269/ajtmh.15-0422 PMC4710429

[9] Mongkolsapaya J, Dejnirattisai W, Xu X-n, Vasanawathana S, Tangthawornchaikul N, et al (2003) Original antigenic sin and apoptosis in the pathogenesis of dengue hemorrhagic fever. Nat Med 9: 921–927. doi: 10.1038/nm887 12808447 10.1038/nm887

[10] Sangkawibha N, Rojanasuphot S, Ahandrik S, Viriyapongse S, Jatanasen S, et al (1984) Risk factors in dengue shock syndrome: a prospective epidemiologic study in Rayong, Thailand. I. The 1980 outbreak. Am J Epidemiol 120: 653–669. 6496446 10.1093/oxfordjournals.aje.a113932

[11] Guzmán MG, Kouri G, Valdes L, Bravo J, Alvarez M, et al (2000) Epidemiologic studies on Dengue in Santiago de Cuba, 1997. Am J Epidemiol 152: 793–799. 11085389 10.1093/aje/152.9.793

[12] Halstead SB (1979) In vivo enhancement of dengue virus infection in rhesus monkeys by passively transferred antibody. J Infect Dis 140: 527–533. 117061 10.1093/infdis/140.4.527

[13] Halstead SB, O'Rourke EJ (1977) Antibody-enhanced dengue virus infection in primate leukocytes. Nature 265: 739–741. 404559 10.1038/265739a0

[14] Guzman MG, Halstead SB, Artsob H, Buchy P, Farrar J, et al (2010) Dengue: a continuing global threat. Nat Rev Microbiol 8: S7–S16. doi: 10.1038/nrmicro2460 21079655 10.1038/nrmicro2460 PMC4333201

[15] Twiddy SS, Holmes EC, Rambaut A (2003) Inferring the rate and time-scale of dengue virus evolution. Mol Biol Evol 20: 122–129. 12519914 10.1093/molbev/msg010

[16] Pybus OG, Tatem AJ, Lemey P (2015) Virus evolution and transmission in an ever more connected world. Proc Biol Sci 282: 20142878 doi: 10.1098/rspb.2014.2878 26702033 10.1098/rspb.2014.2878 PMC4707738

[17] Brownstein JS, Wolfe CJ, Mandl KD (2006) Empirical evidence for the effect of airline travel on inter-regional influenza spread in the United States. PLoS Med 3: e401 doi: 10.1371/journal.pmed.0030401 16968115 10.1371/journal.pmed.0030401 PMC1564183

[18] Bogoch II, Brady OJ, Kraemer M, German M, Creatore MI, et al (2016) Anticipating the international spread of Zika virus from Brazil. Lancet 387: 335–336. doi: 10.1016/S0140-6736(16)00080-5 26777915 10.1016/S0140-6736(16)00080-5 PMC4873159

[19] Brockmann D, Helbing D (2013) The hidden geometry of complex, network-driven contagion phenomena. Science 342: 1337–1342. doi: 10.1126/science.1245200 24337289 10.1126/science.1245200

[20] Lemey P, Rambaut A, Bedford T, Faria N, Bielejec F, et al (2014) Unifying viral genetics and human transportation data to predict the global transmission dynamics of human influenza H3N2. PLoS Pathog 10: e1003932 doi: 10.1371/journal.ppat.1003932 24586153 10.1371/journal.ppat.1003932 PMC3930559

[21] Khan K, Arino J, Hu W, Raposo P, Sears J, et al (2009) Spread of a novel influenza A (H1N1) virus via global airline transportation. N Engl J Med 361: 212–214. doi: 10.1056/NEJMc0904559 19564630 10.1056/NEJMc0904559

[22] Nunes MR, Palacios G, Faria NR, Sousa EC Jr, Pantoja JA, et al (2014) Air travel is associated with intracontinental spread of dengue virus serotypes 1–3 in Brazil. PLoS Negl Trop Dis 8: e2769 doi: 10.1371/journal.pntd.0002769 24743730 10.1371/journal.pntd.0002769 PMC3990485

[23] Katoh K, Standley DM (2013) MAFFT multiple sequence alignment software version 7: improvements in performance and usability. Mol Biol Evol 30: 772–780. doi: 10.1093/molbev/mst010 23329690 10.1093/molbev/mst010 PMC3603318

[24] Martin DP, Lemey P, Lott M, Moulton V, Posada D, et al (2010) RDP3: a flexible and fast computer program for analyzing recombination. Bioinformatics 26: 2462–2463. doi: 10.1093/bioinformatics/btq467 20798170 10.1093/bioinformatics/btq467 PMC2944210

[25] Rambaut A, Lam TT, Carvalho LM, Pybus OG (2016) Exploring the temporal structure of heterochronous sequences using TempEst (formerly Path-O-Gen). Virus Evolution 2: vew007 doi: 10.1093/ve/vew007 27774300 10.1093/ve/vew007 PMC4989882

[26] Minin VN, Bloomquist EW, Suchard MA (2008) Smooth skyride through a rough skyline: Bayesian coalescent-based inference of population dynamics. Mol Biol Evol 25: 1459–1471. doi: 10.1093/molbev/msn090 18408232 10.1093/molbev/msn090 PMC3302198

[27] Drummond AJ, Suchard MA, Xie D, Rambaut A (2012) Bayesian phylogenetics with BEAUti and the BEAST 1.7. Mol Biol Evol 29: 1969–1973. doi: 10.1093/molbev/mss075 22367748 10.1093/molbev/mss075 PMC3408070

[28] Suchard MA, Rambaut A (2009) Many-core algorithms for statistical phylogenetics. Bioinformatics 25: 1370–1376. doi: 10.1093/bioinformatics/btp244 19369496 10.1093/bioinformatics/btp244 PMC2682525

[29] Edwards CJ, Suchard MA, Lemey P, Welch JJ, Barnes I, et al (2011) Ancient hybridization and an Irish origin for the modern polar bear matriline. Curr Biol 21: 1251–1258. doi: 10.1016/j.cub.2011.05.058 21737280 10.1016/j.cub.2011.05.058 PMC4677796

[30] Lemey P, Rambaut A, Drummond AJ, Suchard MA (2009) Bayesian phylogeography finds its roots. PLoS Comput Biol 5: e1000520 doi: 10.1371/journal.pcbi.1000520 19779555 10.1371/journal.pcbi.1000520 PMC2740835

[31] Bielejec F, Rambaut A, Suchard MA, Lemey P (2011) SPREAD: spatial phylogenetic reconstruction of evolutionary dynamics. Bioinformatics 27: 2910–2912. doi: 10.1093/bioinformatics/btr481 21911333 10.1093/bioinformatics/btr481 PMC3187652

[32] Tian H, Zhou S, Dong L, Van Boeckel TP, Cui Y, et al (2015) Avian influenza H5N1 viral and bird migration networks in Asia. Proc Natl Acad Sci USA 112: 172–177. doi: 10.1073/pnas.1405216112 25535385 10.1073/pnas.1405216112 PMC4291667

[33] Minin VN, Suchard MA (2008) Counting labeled transitions in continuous-time Markov models of evolution. J Math Biol 56: 391–412. doi: 10.1007/s00285-007-0120-8 17874105 10.1007/s00285-007-0120-8

[34] Nelson MI, Lemey P, Tan Y, Vincent A, Lam TT-Y, et al (2011) Spatial dynamics of human-origin H1 influenza A virus in North American swine. PLoS Pathog 7: e1002077 doi: 10.1371/journal.ppat.1002077 21695237 10.1371/journal.ppat.1002077 PMC3111536

[35] Faria NR, Suchard MA, Rambaut A, Streicker DG, Lemey P (2013) Simultaneously reconstructing viral cross-species transmission history and identifying the underlying constraints. Philos Trans R Soc Lond B Biol Sci 368: 20120196 doi: 10.1098/rstb.2012.0196 23382420 10.1098/rstb.2012.0196 PMC3678322

[36] Hijmans RJ, Cameron SE, Parra JL, Jones PG, Jarvis A (2005) Very high resolution interpolated climate surfaces for global land areas. Int J Climatol 25: 1965–1978.

[37] Sgouridis S, Bonnefoy PA, Hansman RJ (2011) Air transportation in a carbon constrained world: Long-term dynamics of policies and strategies for mitigating the carbon footprint of commercial aviation. Transp Res Part A Policy Pract 45: 1077–1091.

[38] Pybus OG, Rambaut A (2009) Evolutionary analysis of the dynamics of viral infectious disease. Nat Rev Genet 10: 540–550. doi: 10.1038/nrg2583 19564871 10.1038/nrg2583 PMC7097015

[39] Schreiber MJ, Holmes EC, Ong SH, Soh HS, Liu W, et al (2009) Genomic epidemiology of a dengue virus epidemic in urban Singapore. J Virol 83: 4163–4173. doi: 10.1128/JVI.02445-08 19211734 10.1128/JVI.02445-08 PMC2668455

[40] Tatem AJ, Rogers DJ, Hay SI (2006) Estimating the malaria risk of African mosquito movement by air travel. Malaria journal 5: 57 doi: 10.1186/1475-2875-5-57 16842613 10.1186/1475-2875-5-57 PMC1557515

[41] Gratz NG, Steffen R, Cocksedge W (2000) Why aircraft disinsection? Bull World Health Organ 78: 995–1004. 10994283 PMC2560818

[42] Misao T, Ishihara M (1945) An experiment on the transportation of vector mosquitoes by aircraft (in japanese). Rinsho to Kenkyu 22: 44–46.

[43] Russell RC (1987) Survival of insects in the wheel bays of a Boeing 747B aircraft on flights between tropical and temperate airports. Bull World Health Organ 65: 659–662. 3501345 PMC2491069

[44] Hutchinson R, Bayoh M, Lindsay S (2005) Risk of airport malaria in the UK. European Mosquito Bulletin 19: 12–13.

[45] Schmidt W-P, Suzuki M, Thiem VD, White RG, Tsuzuki A, et al (2011) Population density, water supply, and the risk of dengue fever in Vietnam: cohort study and spatial analysis. PLoS Med 8: e1001082 doi: 10.1371/journal.pmed.1001082 21918642 10.1371/journal.pmed.1001082 PMC3168879

[46] Sang S, Chen B, Wu H, Yang Z, Di B, et al (2015) Dengue is still an imported disease in China: a case study in Guangzhou. Infect Genet Evol 32: 178–190. doi: 10.1016/j.meegid.2015.03.005 25772205 10.1016/j.meegid.2015.03.005

[47] Eisen L, Lozano-Fuentes S (2009) Use of mapping and spatial and space-time modeling approaches in operational control of Aedes aegypti and dengue. PLoS Negl Trop Dis 3: e411 doi: 10.1371/journal.pntd.0000411 19399163 10.1371/journal.pntd.0000411 PMC2668799

[48] Hales S, De Wet N, Maindonald J, Woodward A (2002) Potential effect of population and climate changes on global distribution of dengue fever: an empirical model. Lancet 360: 830–834. doi: 10.1016/S0140-6736(02)09964-6 12243917 10.1016/S0140-6736(02)09964-6

[49] Patz JA, Martens W, Focks DA, Jetten TH (1998) Dengue fever epidemic potential as projected by general circulation models of global climate change. Environ Health Perspect 106: 147–153. 9452414 10.1289/ehp.98106147 PMC1533051

[50] Reeves WC, Hardy JL, Reisen WK, Milby MM (1994) Potential effect of global warming on mosquito-borne arboviruses. J Med Entomol 31: 323–332. 8057305 10.1093/jmedent/31.3.323

[51] Reiter P (2001) Climate change and mosquito-borne disease. Environ Health Perspect 109: 141–161. 11250812 10.1289/ehp.01109s1141 PMC1240549

[52] Nunes MRT, Faria NR, Vasconcelos HB, Medeiros DBdA, Silva de Lima CP, et al (2012) Phylogeography of dengue virus serotype 4, Brazil, 2010–2011. Emerg Infect Dis 18: 1858–1864. doi: 10.3201/eid1811.120217 23092706 10.3201/eid1811.120217 PMC3559147

[53] Pybus OG, Suchard MA, Lemey P, Bernardin FJ, Rambaut A, et al (2012) Unifying the spatial epidemiology and molecular evolution of emerging epidemics. Proc Natl Acad Sci USA 109: 15066–15071. doi: 10.1073/pnas.1206598109 22927414 10.1073/pnas.1206598109 PMC3443149

[54] Coudeville L, Baurin N, L’Azou M, Guy B (2016) Potential impact of dengue vaccination: Insights from two large-scale phase III trials with a tetravalent dengue vaccine. Vaccine 34: 6426–6435. doi: 10.1016/j.vaccine.2016.08.050 27601343 10.1016/j.vaccine.2016.08.050

