# Supplementary figures and images for: Increasing airline travel may facilitate co-circulation of multiple dengue virus serotypes in Asia
Source: PLoS Negl Trop Dis. 2017 Aug 3;11(8):e0005694. doi: 10.1371/journal.pntd.0005694 (PMC5542384; doi:10.1371/journal.pntd.0005694)

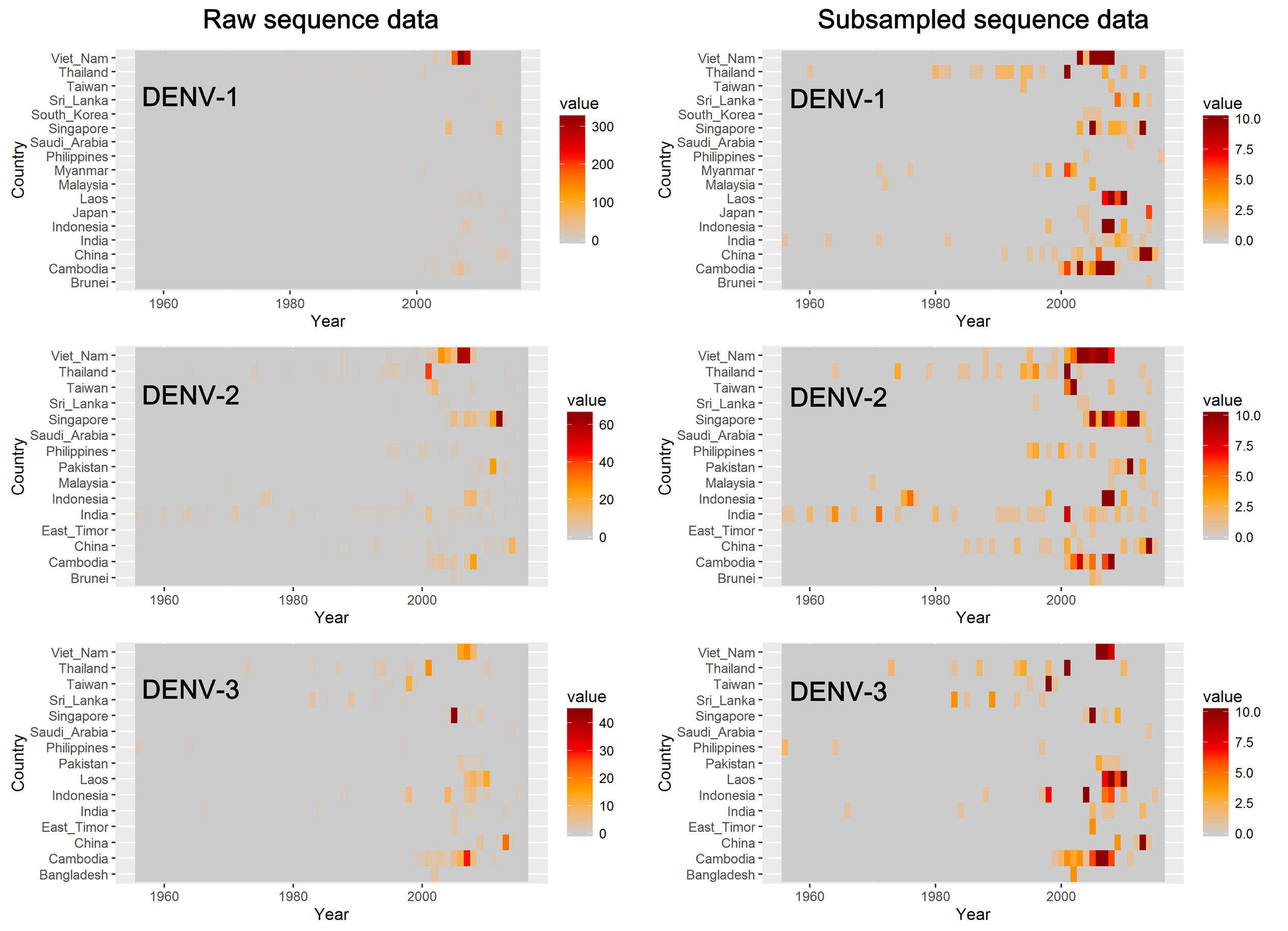

Supplement: S1 Fig — The color spectrum shows the number of sequences from each year and location, from grey (low numbers of sequences) to dark red (high numbers of sequences). The raw data set (left column) contained 1272 DENV-1 sequences, 628 DENV-2 sequences and 302 DENV-3 sequences. After randomly subsampling by location and sampling time (right column) the total number of subsampled sequences analyzed were 327 for DENV-1, 357 for DENV-2, and 202 for DENV-3. (TIF) [file pntd.0005694.s001.tif]

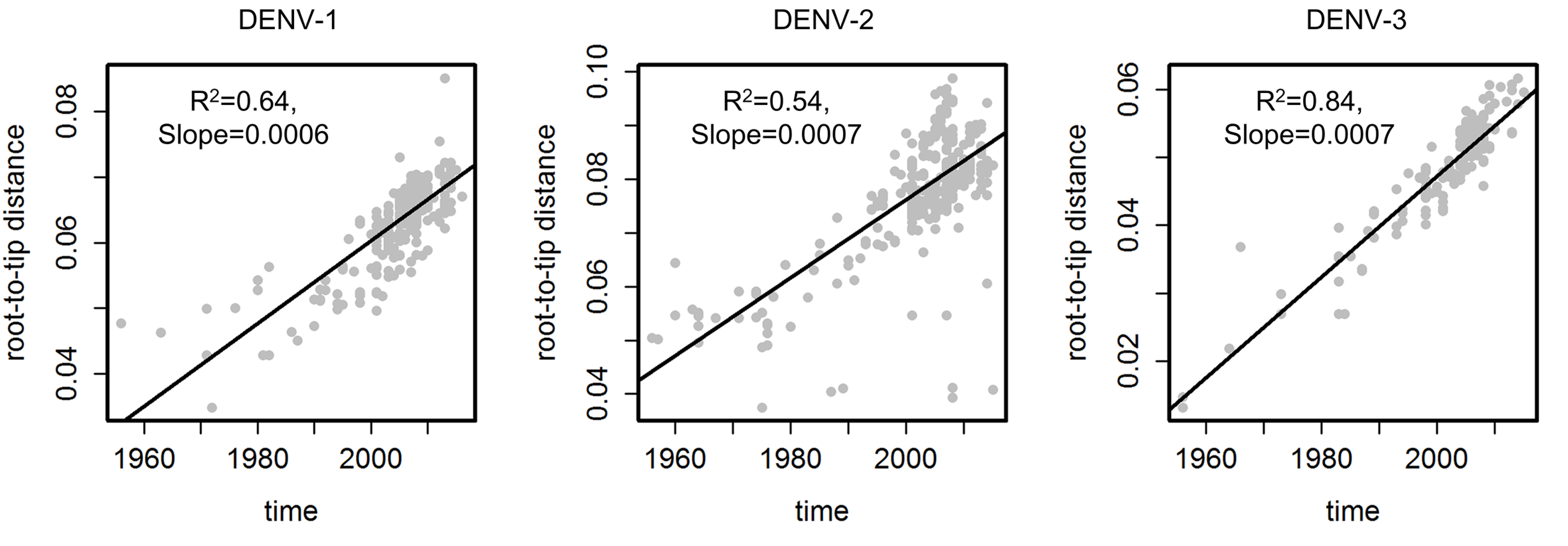

Supplement: S2 Fig — TempEst was used to obtain exploratory regressions based on the maximum likelihood trees. (TIF) [file pntd.0005694.s002.tif]

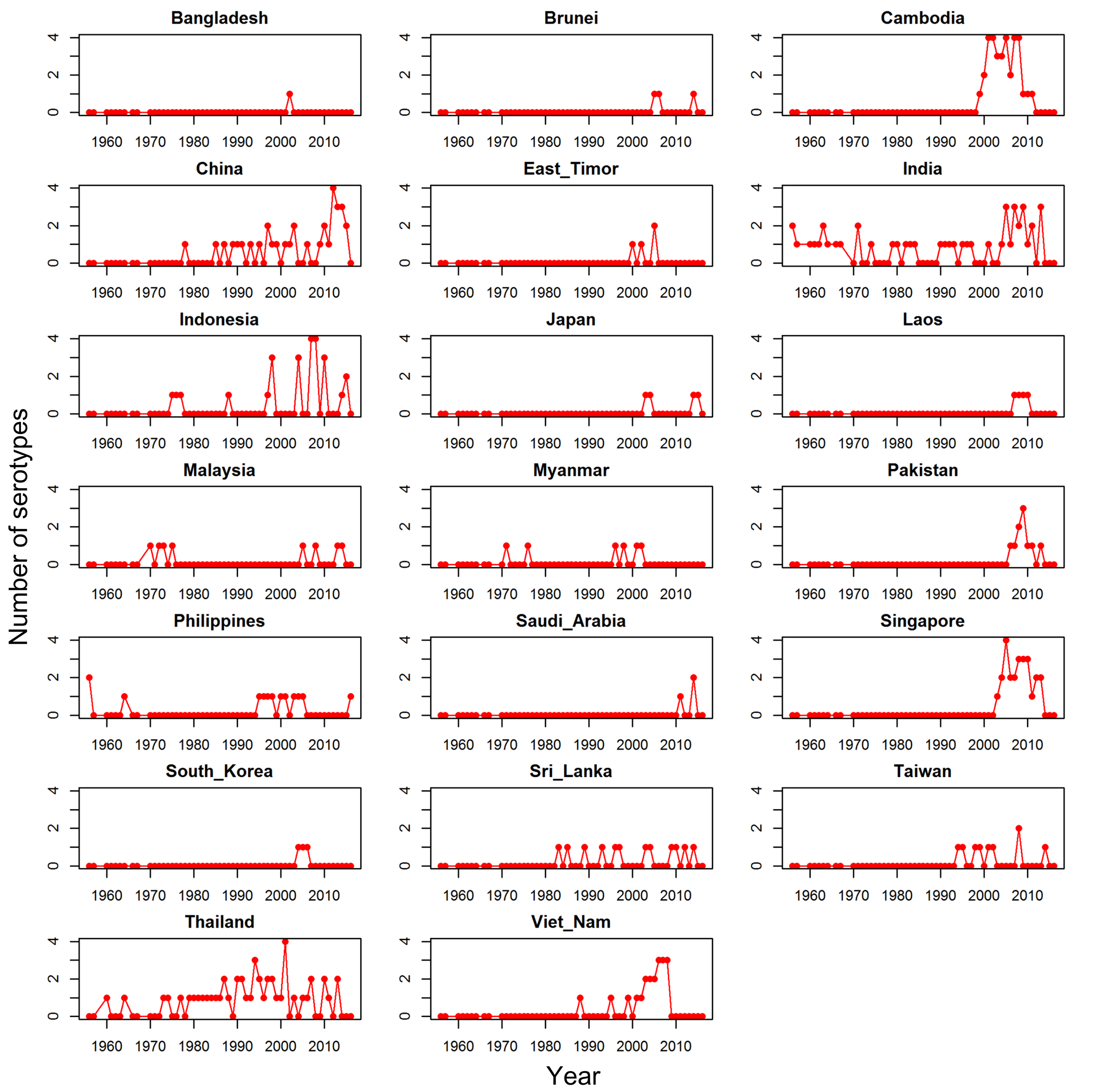

Supplement: S3 Fig — (TIF) [file pntd.0005694.s003.tif]

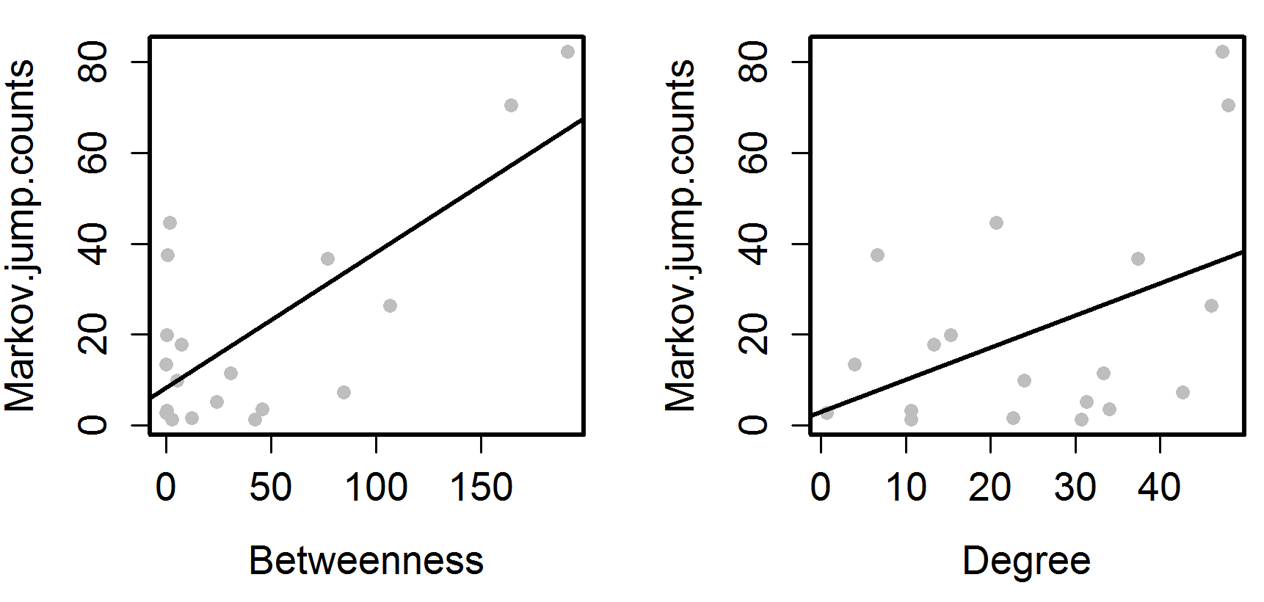

Supplement: S4 Fig — (TIFF) [file pntd.0005694.s004.tiff]

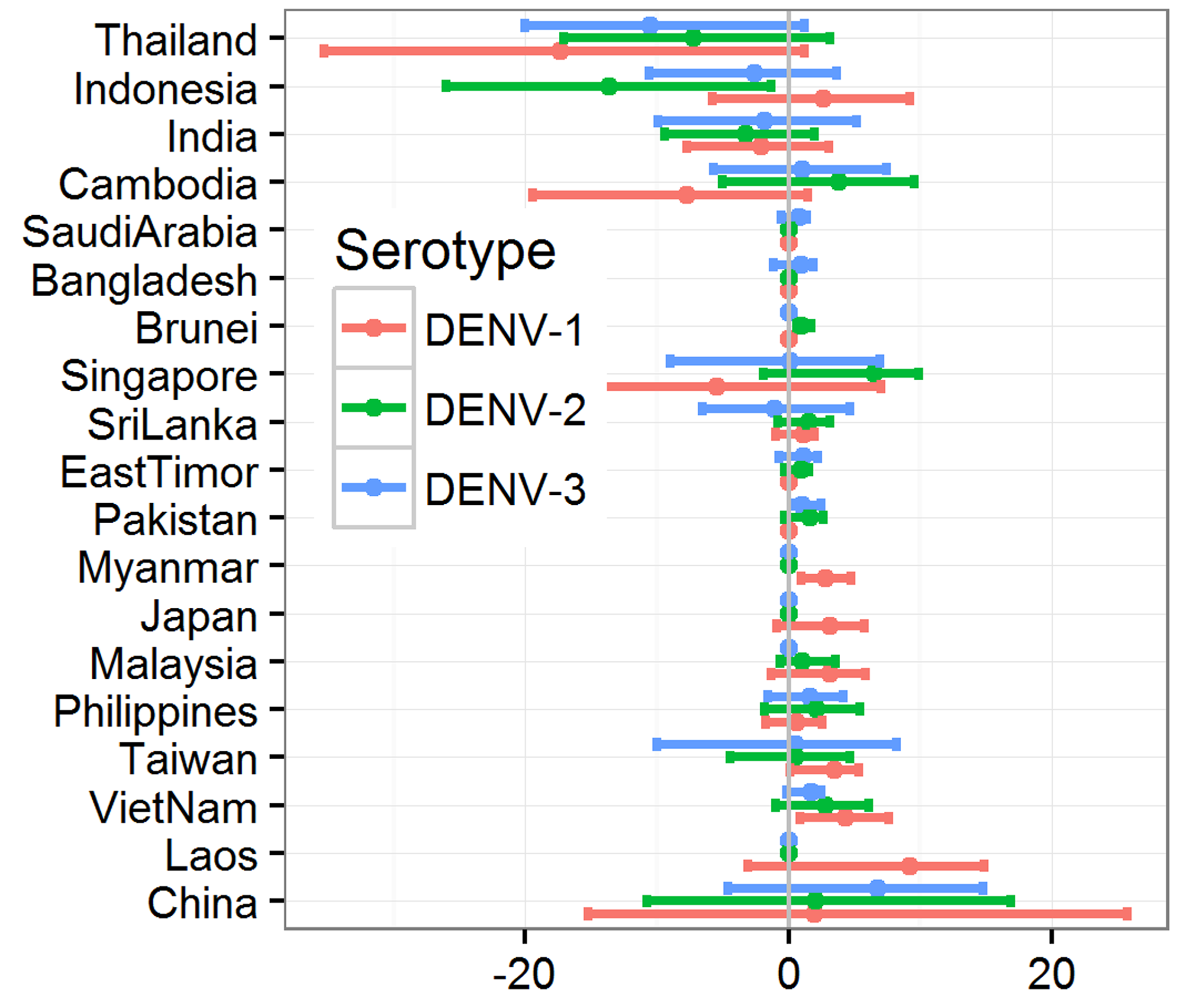

Supplement: S5 Fig — For each country, we summarize the average net Markov jumps (jumps to—jumps from) and their 95% credible intervals. The estimates are reordered from the lowest to highest net jumps. (TIF) [file pntd.0005694.s005.tif]

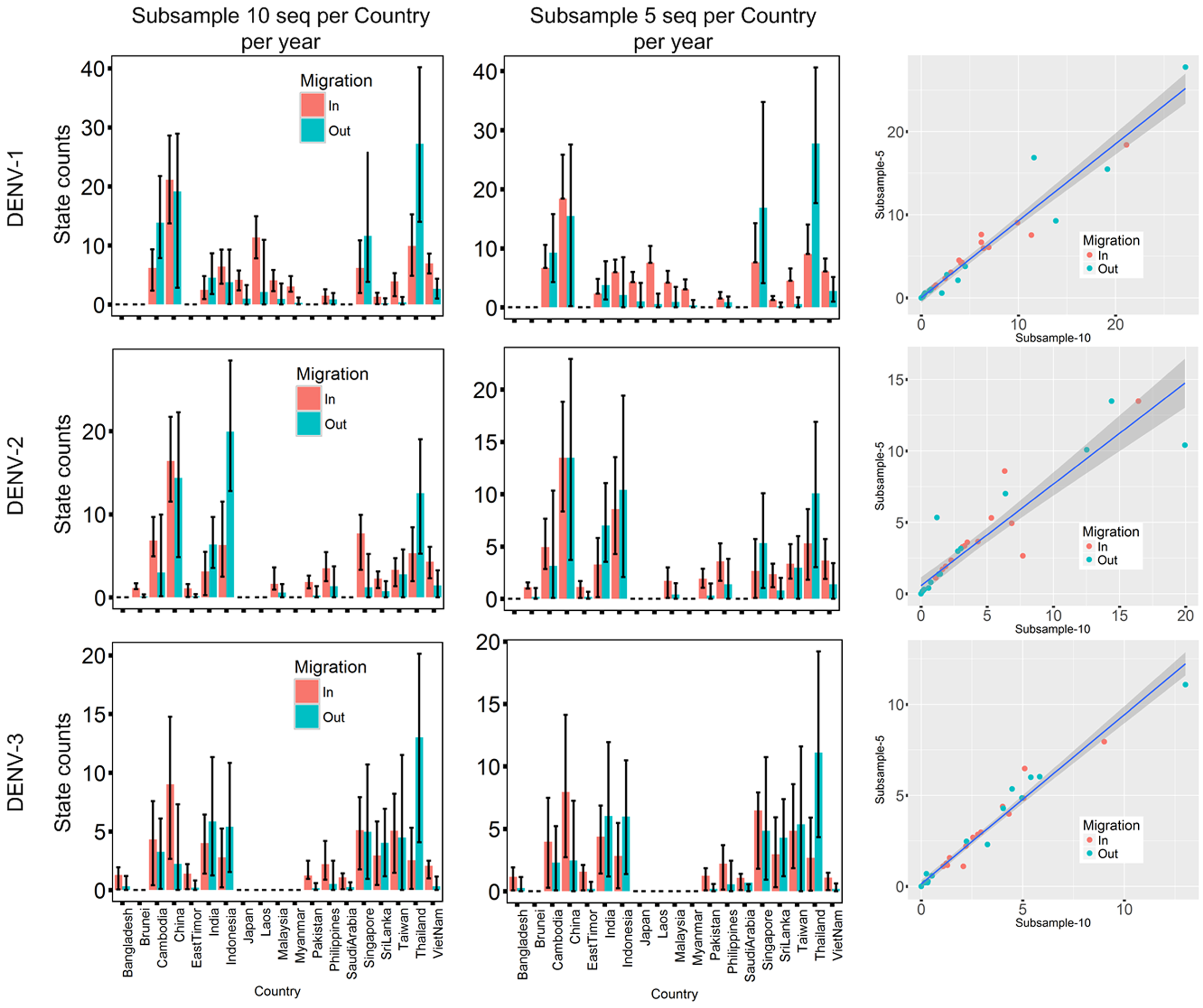

Supplement: S6 Fig — Error bars represent 95% highest posterior density intervals. Left panel: at most 10 sequences were sampled per country per year. Middle panel: at most 5 sequences were sampled per country per year. Right panel: scatter plot of state transitions of the two subsampled data sets. (TIF) [file pntd.0005694.s006.tif]
