# Supplementary material for: Increasing airline travel may facilitate co-circulation of multiple dengue virus serotypes in Asia
Source: PLoS Negl Trop Dis. 2017 Aug 3;11(8):e0005694. doi: 10.1371/journal.pntd.0005694 (PMC5542384; doi:10.1371/journal.pntd.0005694)
Supplement: S1 Table — (DOCX) [file pntd.0005694.s007.docx]

**S1 Table** Subsampled DENV E gene sequences in study

| Accession number | Location | Year | Serotype |
| --- | --- | --- | --- |
| AB189120 | Indonesia | 1998 | DENV-1 |
| AB189121 | Indonesia | 1998 | DENV-1 |
| AB195673 | Japan | 2003 | DENV-1 |
| AB204803 | Japan | 2004 | DENV-1 |
| AB608786 | Taiwan | 2008 | DENV-1 |
| AB608787 | Taiwan | 2008 | DENV-1 |
| AB608788 | Taiwan | 1994 | DENV-1 |
| AB608789 | Taiwan | 1994 | DENV-1 |
| AY713473 | Myanmar | 1971 | DENV-1 |
| AY713474 | Myanmar | 2001 | DENV-1 |
| AY713475 | Myanmar | 2001 | DENV-1 |
| AY713476 | Myanmar | 2001 | DENV-1 |
| AY722801 | Myanmar | 1976 | DENV-1 |
| AY722802 | Myanmar | 1996 | DENV-1 |
| AY722803 | Myanmar | 1998 | DENV-1 |
| AY726549 | Myanmar | 2001 | DENV-1 |
| AY726550 | Myanmar | 2001 | DENV-1 |
| AY726551 | Myanmar | 2001 | DENV-1 |
| AY726552 | Myanmar | 2002 | DENV-1 |
| AY726553 | Myanmar | 2002 | DENV-1 |
| AY726554 | Myanmar | 1998 | DENV-1 |
| AY726555 | Myanmar | 1998 | DENV-1 |
| AY732474 | Thailand | 1980 | DENV-1 |
| AY732475 | Thailand | 1994 | DENV-1 |
| AY732476 | Thailand | 1980 | DENV-1 |
| AY732477 | Thailand | 1991 | DENV-1 |
| AY732478 | Thailand | 1991 | DENV-1 |
| AY732479 | Thailand | 2001 | DENV-1 |
| AY732480 | Thailand | 1994 | DENV-1 |
| AY732481 | Thailand | 1982 | DENV-1 |
| AY732482 | Thailand | 2001 | DENV-1 |
| AY732483 | Thailand | 1981 | DENV-1 |
| AY858983 | Indonesia | 2004 | DENV-1 |
| EF113153 | China | 2006 | DENV-1 |
| EF457905 | Malaysia | 1972 | DENV-1 |
| EU081228 | Singapore | 2005 | DENV-1 |
| EU081231 | Singapore | 2005 | DENV-1 |
| EU081245 | Singapore | 2005 | DENV-1 |
| EU081247 | Singapore | 2005 | DENV-1 |
| EU081253 | Singapore | 2005 | DENV-1 |
| EU081255 | Singapore | 2005 | DENV-1 |
| EU081257 | Singapore | 2005 | DENV-1 |
| EU081262 | Singapore | 2005 | DENV-1 |
| EU081266 | Singapore | 2005 | DENV-1 |
| EU081278 | Singapore | 2005 | DENV-1 |
| EU081280 | Singapore | 2006 | DENV-1 |
| EU081281 | Singapore | 2006 | DENV-1 |
| EU482476 | VietNam | 2003 | DENV-1 |
| EU482528 | VietNam | 2006 | DENV-1 |
| EU482708 | VietNam | 2007 | DENV-1 |
| EU482790 | VietNam | 2003 | DENV-1 |
| EU482791 | VietNam | 2003 | DENV-1 |
| EU482792 | VietNam | 2003 | DENV-1 |
| EU482797 | VietNam | 2006 | DENV-1 |
| EU677163 | VietNam | 2007 | DENV-1 |
| EU677170 | VietNam | 2007 | DENV-1 |
| EU677178 | VietNam | 2007 | DENV-1 |
| FJ024431 | VietNam | 2007 | DENV-1 |
| FJ024460 | VietNam | 2007 | DENV-1 |
| FJ176779 | China | 2006 | DENV-1 |
| FJ176780 | China | 2006 | DENV-1 |
| FJ182023 | VietNam | 2007 | DENV-1 |
| FJ196841 | China | 2003 | DENV-1 |
| FJ196842 | China | 2003 | DENV-1 |
| FJ196844 | China | 2006 | DENV-1 |
| FJ196845 | China | 1991 | DENV-1 |
| FJ196846 | China | 1995 | DENV-1 |
| FJ410279 | VietNam | 2008 | DENV-1 |
| FJ469907 | Singapore | 2003 | DENV-1 |
| FJ469908 | Singapore | 2003 | DENV-1 |
| FJ469909 | Singapore | 2003 | DENV-1 |
| FJ547060 | VietNam | 2007 | DENV-1 |
| FJ639669 | Cambodia | 2000 | DENV-1 |
| FJ639670 | Cambodia | 2001 | DENV-1 |
| FJ639671 | Cambodia | 2001 | DENV-1 |
| FJ639672 | Cambodia | 2001 | DENV-1 |
| FJ639673 | Cambodia | 2001 | DENV-1 |
| FJ639674 | Cambodia | 2002 | DENV-1 |
| FJ639675 | Cambodia | 2003 | DENV-1 |
| FJ639676 | Cambodia | 2003 | DENV-1 |
| FJ639677 | Cambodia | 2003 | DENV-1 |
| FJ639678 | Cambodia | 2003 | DENV-1 |
| FJ639679 | Cambodia | 2003 | DENV-1 |
| FJ639680 | Cambodia | 2003 | DENV-1 |
| FJ639681 | Cambodia | 2003 | DENV-1 |
| FJ639682 | Cambodia | 2004 | DENV-1 |
| FJ639683 | Cambodia | 2005 | DENV-1 |
| FJ639684 | Cambodia | 2005 | DENV-1 |
| FJ639685 | Cambodia | 2005 | DENV-1 |
| FJ639688 | Cambodia | 2007 | DENV-1 |
| FJ639692 | Cambodia | 2007 | DENV-1 |
| FJ639693 | Cambodia | 2007 | DENV-1 |
| FJ687426 | Thailand | 2001 | DENV-1 |
| FJ687427 | Thailand | 2001 | DENV-1 |
| FJ687428 | Thailand | 2001 | DENV-1 |
| FJ687429 | Thailand | 2001 | DENV-1 |
| FJ687430 | Thailand | 2001 | DENV-1 |
| FJ687431 | Thailand | 2001 | DENV-1 |
| FJ687432 | Thailand | 2001 | DENV-1 |
| FJ687433 | Thailand | 2001 | DENV-1 |
| FJ882529 | VietNam | 2006 | DENV-1 |
| FJ882530 | VietNam | 2006 | DENV-1 |
| FJ882536 | VietNam | 2006 | DENV-1 |
| FJ882563 | VietNam | 2003 | DENV-1 |
| FJ882564 | VietNam | 2003 | DENV-1 |
| FJ882565 | VietNam | 2003 | DENV-1 |
| FJ882567 | VietNam | 2003 | DENV-1 |
| FJ882569 | VietNam | 2004 | DENV-1 |
| FJ898386 | VietNam | 2005 | DENV-1 |
| FJ898388 | VietNam | 2005 | DENV-1 |
| FJ898389 | VietNam | 2005 | DENV-1 |
| FJ898390 | VietNam | 2005 | DENV-1 |
| FJ898391 | VietNam | 2005 | DENV-1 |
| FJ898393 | VietNam | 2005 | DENV-1 |
| FJ898395 | VietNam | 2005 | DENV-1 |
| FJ898402 | VietNam | 2006 | DENV-1 |
| FJ898412 | VietNam | 2006 | DENV-1 |
| GQ199772 | VietNam | 2006 | DENV-1 |
| GQ199817 | VietNam | 2007 | DENV-1 |
| GQ199830 | VietNam | 2003 | DENV-1 |
| GQ199832 | VietNam | 2003 | DENV-1 |
| GQ199833 | VietNam | 2004 | DENV-1 |
| GQ199834 | VietNam | 2005 | DENV-1 |
| GQ199835 | VietNam | 2005 | DENV-1 |
| GQ199836 | VietNam | 2005 | DENV-1 |
| GQ199846 | VietNam | 2006 | DENV-1 |
| GQ398255 | Singapore | 2008 | DENV-1 |
| GQ868602 | Philippines | 2004 | DENV-1 |
| GQ868618 | Cambodia | 2003 | DENV-1 |
| GQ868619 | Cambodia | 2003 | DENV-1 |
| GQ868632 | Cambodia | 2008 | DENV-1 |
| GQ868633 | Cambodia | 2008 | DENV-1 |
| GQ868635 | Cambodia | 2008 | DENV-1 |
| GQ868636 | Cambodia | 2008 | DENV-1 |
| GQ868637 | Cambodia | 2000 | DENV-1 |
| GU131699 | VietNam | 2008 | DENV-1 |
| GU131702 | VietNam | 2008 | DENV-1 |
| GU131705 | VietNam | 2008 | DENV-1 |
| GU131799 | VietNam | 2008 | DENV-1 |
| GU131811 | VietNam | 2008 | DENV-1 |
| GU131831 | VietNam | 2008 | DENV-1 |
| GU131887 | Cambodia | 2006 | DENV-1 |
| GU131889 | Cambodia | 2006 | DENV-1 |
| GU131891 | Cambodia | 2006 | DENV-1 |
| GU131892 | Cambodia | 2006 | DENV-1 |
| GU131894 | Cambodia | 2008 | DENV-1 |
| GU131895 | Cambodia | 2009 | DENV-1 |
| GU131919 | Cambodia | 2008 | DENV-1 |
| GU131920 | Cambodia | 2008 | DENV-1 |
| GU131921 | Cambodia | 2008 | DENV-1 |
| GU131922 | Cambodia | 2008 | DENV-1 |
| GU131923 | Cambodia | 2005 | DENV-1 |
| GU370048 | Singapore | 2008 | DENV-1 |
| GU370049 | Singapore | 2008 | DENV-1 |
| HG316481 | Thailand | 2010 | DENV-1 |
| HG316482 | Thailand | 2010 | DENV-1 |
| HM181937 | Cambodia | 2006 | DENV-1 |
| HM181938 | Cambodia | 2006 | DENV-1 |
| HM181939 | Cambodia | 2006 | DENV-1 |
| HM181940 | Cambodia | 2006 | DENV-1 |
| HM181941 | Cambodia | 2006 | DENV-1 |
| HM181947 | Cambodia | 2007 | DENV-1 |
| HM181953 | Cambodia | 2007 | DENV-1 |
| HM181968 | VietNam | 2008 | DENV-1 |
| HM469966 | Thailand | 2007 | DENV-1 |
| HM469967 | Thailand | 2007 | DENV-1 |
| HM469968 | Thailand | 2007 | DENV-1 |
| HM488255 | Cambodia | 2007 | DENV-1 |
| HM631852 | Cambodia | 2006 | DENV-1 |
| HQ624984 | Cambodia | 2007 | DENV-1 |
| HQ891313 | SriLanka | 2009 | DENV-1 |
| HQ891314 | SriLanka | 2009 | DENV-1 |
| HQ891315 | SriLanka | 2009 | DENV-1 |
| HQ891316 | SriLanka | 2009 | DENV-1 |
| JF937599 | VietNam | 2006 | DENV-1 |
| JF937609 | VietNam | 2008 | DENV-1 |
| JF937651 | Cambodia | 2008 | DENV-1 |
| JN054255 | SriLanka | 2010 | DENV-1 |
| JN054256 | SriLanka | 2009 | DENV-1 |
| JN205310 | China | 2002 | DENV-1 |
| JN380803 | Singapore | 2009 | DENV-1 |
| JN380804 | Singapore | 2009 | DENV-1 |
| JN380805 | Singapore | 2009 | DENV-1 |
| JN380806 | Singapore | 2010 | DENV-1 |
| JN380807 | Singapore | 2010 | DENV-1 |
| JN638336 | Thailand | 1986 | DENV-1 |
| JN638337 | Thailand | 1990 | DENV-1 |
| JN638338 | Thailand | 1992 | DENV-1 |
| JN638339 | Thailand | 1995 | DENV-1 |
| JN638340 | Thailand | 1997 | DENV-1 |
| JN638341 | Thailand | 1987 | DENV-1 |
| JN638342 | Thailand | 1990 | DENV-1 |
| JN638343 | Thailand | 1992 | DENV-1 |
| JN638344 | Thailand | 1995 | DENV-1 |
| JN697056 | Malaysia | 2005 | DENV-1 |
| JN697057 | Malaysia | 2005 | DENV-1 |
| JN697058 | Malaysia | 2005 | DENV-1 |
| JN819423 | Cambodia | 2001 | DENV-1 |
| JN903578 | India | 2007 | DENV-1 |
| JN903579 | India | 2008 | DENV-1 |
| JN903580 | India | 2009 | DENV-1 |
| JN903581 | India | 2009 | DENV-1 |
| JQ048541 | China | 2011 | DENV-1 |
| JQ287662 | VietNam | 2007 | DENV-1 |
| JQ287664 | Cambodia | 2004 | DENV-1 |
| JQ692085 | India | 2010 | DENV-1 |
| JQ917404 | India | 2009 | DENV-1 |
| JQ922544 | India | 1963 | DENV-1 |
| JQ922545 | India | 1982 | DENV-1 |
| JQ922546 | India | 1971 | DENV-1 |
| JQ922548 | India | 2005 | DENV-1 |
| KC172829 | Laos | 2008 | DENV-1 |
| KC172830 | Laos | 2008 | DENV-1 |
| KC172831 | Laos | 2008 | DENV-1 |
| KC172832 | Laos | 2009 | DENV-1 |
| KC172833 | Laos | 2008 | DENV-1 |
| KC172834 | Laos | 2008 | DENV-1 |
| KC172835 | Laos | 2008 | DENV-1 |
| KC182083 | Laos | 2010 | DENV-1 |
| KC182084 | Laos | 2010 | DENV-1 |
| KC182086 | Laos | 2009 | DENV-1 |
| KC182087 | Laos | 2009 | DENV-1 |
| KC182088 | Laos | 2009 | DENV-1 |
| KC182089 | Laos | 2009 | DENV-1 |
| KC182090 | Laos | 2009 | DENV-1 |
| KC182091 | Laos | 2010 | DENV-1 |
| KC182092 | Laos | 2010 | DENV-1 |
| KC182093 | Laos | 2010 | DENV-1 |
| KC182095 | Laos | 2010 | DENV-1 |
| KC182096 | Laos | 2007 | DENV-1 |
| KC182097 | Laos | 2007 | DENV-1 |
| KC182098 | Laos | 2007 | DENV-1 |
| KC182099 | Laos | 2007 | DENV-1 |
| KC182100 | Laos | 2007 | DENV-1 |
| KC182101 | Laos | 2007 | DENV-1 |
| KC182102 | Laos | 2007 | DENV-1 |
| KC182104 | Laos | 2008 | DENV-1 |
| KC182105 | Laos | 2008 | DENV-1 |
| KC182106 | Laos | 2008 | DENV-1 |
| KC182107 | Laos | 2008 | DENV-1 |
| KC182109 | Laos | 2010 | DENV-1 |
| KC182111 | Laos | 2010 | DENV-1 |
| KC182112 | Laos | 2010 | DENV-1 |
| KC182113 | Laos | 2010 | DENV-1 |
| KC759167 | China | 2012 | DENV-1 |
| KC762621 | Indonesia | 2008 | DENV-1 |
| KC762622 | Indonesia | 2008 | DENV-1 |
| KC762623 | Indonesia | 2007 | DENV-1 |
| KC762625 | Indonesia | 2007 | DENV-1 |
| KC762627 | Indonesia | 2008 | DENV-1 |
| KC762628 | Indonesia | 2008 | DENV-1 |
| KC762630 | Indonesia | 2008 | DENV-1 |
| KC762632 | Indonesia | 2007 | DENV-1 |
| KC762635 | Indonesia | 2007 | DENV-1 |
| KC762636 | Indonesia | 2007 | DENV-1 |
| KC762638 | Indonesia | 2008 | DENV-1 |
| KC762639 | Indonesia | 2010 | DENV-1 |
| KC762641 | Indonesia | 2008 | DENV-1 |
| KC762642 | Indonesia | 2010 | DENV-1 |
| KC762643 | Indonesia | 2008 | DENV-1 |
| KC762644 | Indonesia | 2008 | DENV-1 |
| KC762646 | Indonesia | 2007 | DENV-1 |
| KC762647 | Indonesia | 2010 | DENV-1 |
| KC762649 | Indonesia | 2007 | DENV-1 |
| KC762650 | Indonesia | 2007 | DENV-1 |
| KC762651 | Indonesia | 2007 | DENV-1 |
| KC762653 | Indonesia | 2008 | DENV-1 |
| KC762654 | Indonesia | 2007 | DENV-1 |
| KC863940 | India | 2010 | DENV-1 |
| KF289072 | India | 2011 | DENV-1 |
| KF289073 | India | 1956 | DENV-1 |
| KF864667 | China | 2013 | DENV-1 |
| KF887994 | Thailand | 2013 | DENV-1 |
| KF921932 | Cambodia | 2007 | DENV-1 |
| KF921933 | Cambodia | 2007 | DENV-1 |
| KF921942 | VietNam | 2008 | DENV-1 |
| KF955406 | Cambodia | 2001 | DENV-1 |
| KF955440 | Cambodia | 2007 | DENV-1 |
| KF955445 | Cambodia | 2003 | DENV-1 |
| KF971869 | China | 2013 | DENV-1 |
| KF971871 | China | 2013 | DENV-1 |
| KJ438293 | China | 2013 | DENV-1 |
| KJ438296 | China | 2013 | DENV-1 |
| KJ545449 | China | 2013 | DENV-1 |
| KJ545453 | China | 2013 | DENV-1 |
| KJ545454 | China | 2013 | DENV-1 |
| KJ545460 | China | 2013 | DENV-1 |
| KJ545461 | China | 2013 | DENV-1 |
| KJ545482 | Thailand | 2013 | DENV-1 |
| KJ726662 | SriLanka | 2012 | DENV-1 |
| KJ726663 | SriLanka | 2012 | DENV-1 |
| KJ726664 | SriLanka | 2012 | DENV-1 |
| KJ726665 | SriLanka | 2012 | DENV-1 |
| KJ755855 | India | 2013 | DENV-1 |
| KJ933413 | China | 2012 | DENV-1 |
| KM403575 | Singapore | 2012 | DENV-1 |
| KM403576 | Singapore | 2013 | DENV-1 |
| KM403577 | Singapore | 2013 | DENV-1 |
| KM403578 | Singapore | 2013 | DENV-1 |
| KM403589 | Singapore | 2013 | DENV-1 |
| KM403590 | Singapore | 2013 | DENV-1 |
| KM403596 | Singapore | 2013 | DENV-1 |
| KM403605 | Singapore | 2013 | DENV-1 |
| KM403614 | Singapore | 2013 | DENV-1 |
| KM403627 | Singapore | 2013 | DENV-1 |
| KM403632 | Singapore | 2013 | DENV-1 |
| KP398852 | SriLanka | 2014 | DENV-1 |
| KP686070 | China | 2014 | DENV-1 |
| KP723473 | China | 2014 | DENV-1 |
| KP723476 | China | 2014 | DENV-1 |
| KP772252 | China | 2014 | DENV-1 |
| KR024708 | China | 2014 | DENV-1 |
| KR028435 | China | 2014 | DENV-1 |
| KR071622 | China | 2014 | DENV-1 |
| KT187559 | China | 2014 | DENV-1 |
| KT187560 | China | 2014 | DENV-1 |
| KT187564 | China | 2014 | DENV-1 |
| KT306907 | China | 2015 | DENV-1 |
| KT831765 | Indonesia | 2014 | DENV-1 |
| LC011945 | Japan | 2014 | DENV-1 |
| LC011946 | Japan | 2014 | DENV-1 |
| LC011947 | Japan | 2014 | DENV-1 |
| LC011948 | Japan | 2014 | DENV-1 |
| LC011949 | Japan | 2014 | DENV-1 |
| LC016760 | Japan | 2014 | DENV-1 |
| LC128301 | Philippines | 2016 | DENV-1 |
| EU179857 | Brunei | 2005 | DENV-2 |
| EU179858 | Brunei | 2005 | DENV-2 |
| JF730044 | Cambodia | 2001 | DENV-2 |
| FJ639697 | Cambodia | 2001 | DENV-2 |
| FJ639698 | Cambodia | 2002 | DENV-2 |
| FJ639699 | Cambodia | 2002 | DENV-2 |
| FJ639700 | Cambodia | 2002 | DENV-2 |
| FJ639701 | Cambodia | 2002 | DENV-2 |
| KF921930 | Cambodia | 2002 | DENV-2 |
| KF921931 | Cambodia | 2003 | DENV-2 |
| GQ868620 | Cambodia | 2003 | DENV-2 |
| GQ868621 | Cambodia | 2003 | DENV-2 |
| GQ868622 | Cambodia | 2003 | DENV-2 |
| FJ639702 | Cambodia | 2003 | DENV-2 |
| FJ639703 | Cambodia | 2003 | DENV-2 |
| FJ639704 | Cambodia | 2003 | DENV-2 |
| FJ639705 | Cambodia | 2003 | DENV-2 |
| FJ639706 | Cambodia | 2004 | DENV-2 |
| FJ639707 | Cambodia | 2004 | DENV-2 |
| GQ868623 | Cambodia | 2005 | DENV-2 |
| FJ639708 | Cambodia | 2005 | DENV-2 |
| FJ639709 | Cambodia | 2005 | DENV-2 |
| FJ639710 | Cambodia | 2005 | DENV-2 |
| FJ639711 | Cambodia | 2005 | DENV-2 |
| GQ868624 | Cambodia | 2007 | DENV-2 |
| GU131927 | Cambodia | 2007 | DENV-2 |
| GU131896 | Cambodia | 2007 | DENV-2 |
| GU131897 | Cambodia | 2007 | DENV-2 |
| JN368476 | Cambodia | 2007 | DENV-2 |
| FJ639717 | Cambodia | 2007 | DENV-2 |
| JF730046 | Cambodia | 2008 | DENV-2 |
| JF730047 | Cambodia | 2008 | DENV-2 |
| GU131924 | Cambodia | 2008 | DENV-2 |
| GU131901 | Cambodia | 2008 | DENV-2 |
| GU131929 | Cambodia | 2008 | DENV-2 |
| GU131902 | Cambodia | 2008 | DENV-2 |
| GU131931 | Cambodia | 2008 | DENV-2 |
| GQ868625 | Cambodia | 2008 | DENV-2 |
| GQ868631 | Cambodia | 2008 | DENV-2 |
| KF955399 | Cambodia | 2008 | DENV-2 |
| AF119661 | China | 1985 | DENV-2 |
| AF204178 | China | 1987 | DENV-2 |
| AF204177 | China | 1989 | DENV-2 |
| KC964094 | China | 1993 | DENV-2 |
| FJ196854 | China | 1993 | DENV-2 |
| FJ196851 | China | 1998 | DENV-2 |
| KC964095 | China | 1998 | DENV-2 |
| KC964093 | China | 2001 | DENV-2 |
| EF051521 | China | 2001 | DENV-2 |
| FJ196852 | China | 2001 | DENV-2 |
| FJ196853 | China | 2003 | DENV-2 |
| JX470186 | China | 2010 | DENV-2 |
| KP723479 | China | 2010 | DENV-2 |
| KC131142 | China | 2012 | DENV-2 |
| KJ545451 | China | 2013 | DENV-2 |
| KJ545457 | China | 2013 | DENV-2 |
| KF479233 | China | 2013 | DENV-2 |
| KT187554 | China | 2014 | DENV-2 |
| KT187555 | China | 2014 | DENV-2 |
| KT187558 | China | 2014 | DENV-2 |
| KP723478 | China | 2014 | DENV-2 |
| KP191524 | China | 2014 | DENV-2 |
| KP191525 | China | 2014 | DENV-2 |
| KP191527 | China | 2014 | DENV-2 |
| KP191528 | China | 2014 | DENV-2 |
| KP191529 | China | 2014 | DENV-2 |
| KP191530 | China | 2014 | DENV-2 |
| KU094070 | China | 2015 | DENV-2 |
| AY706016 | EastTimor | 2002 | DENV-2 |
| LC111438 | EastTimor | 2005 | DENV-2 |
| FJ538926 | India | 1956 | DENV-2 |
| FJ538927 | India | 1957 | DENV-2 |
| FJ538928 | India | 1960 | DENV-2 |
| JQ922552 | India | 1960 | DENV-2 |
| FJ538914 | India | 1963 | DENV-2 |
| FJ538907 | India | 1964 | DENV-2 |
| FJ538908 | India | 1964 | DENV-2 |
| FJ538909 | India | 1964 | DENV-2 |
| FJ538910 | India | 1964 | DENV-2 |
| FJ538915 | India | 1967 | DENV-2 |
| FJ538916 | India | 1971 | DENV-2 |
| FJ538917 | India | 1971 | DENV-2 |
| FJ538918 | India | 1971 | DENV-2 |
| FJ538919 | India | 1971 | DENV-2 |
| FJ538920 | India | 1974 | DENV-2 |
| FJ538921 | India | 1980 | DENV-2 |
| FJ538922 | India | 1983 | DENV-2 |
| FJ538924 | India | 1990 | DENV-2 |
| FJ538923 | India | 1991 | DENV-2 |
| FJ538925 | India | 1992 | DENV-2 |
| FJ538911 | India | 1993 | DENV-2 |
| FJ538912 | India | 1995 | DENV-2 |
| JQ922549 | India | 1996 | DENV-2 |
| FJ538913 | India | 1997 | DENV-2 |
| DQ448232 | India | 2001 | DENV-2 |
| DQ448233 | India | 2001 | DENV-2 |
| DQ448234 | India | 2001 | DENV-2 |
| DQ448235 | India | 2001 | DENV-2 |
| DQ448236 | India | 2001 | DENV-2 |
| DQ448237 | India | 2001 | DENV-2 |
| DQ448238 | India | 2001 | DENV-2 |
| DQ448231 | India | 2001 | DENV-2 |
| FJ538905 | India | 2004 | DENV-2 |
| FJ538906 | India | 2005 | DENV-2 |
| FJ898454 | India | 2006 | DENV-2 |
| KJ918750 | India | 2007 | DENV-2 |
| JQ955623 | India | 2009 | DENV-2 |
| JX475906 | India | 2009 | DENV-2 |
| JQ955624 | India | 2011 | DENV-2 |
| KJ545478 | India | 2013 | DENV-2 |
| GQ398258 | Indonesia | 1975 | DENV-2 |
| GQ398263 | Indonesia | 1975 | DENV-2 |
| GQ398268 | Indonesia | 1975 | DENV-2 |
| GQ398264 | Indonesia | 1976 | DENV-2 |
| GQ398259 | Indonesia | 1976 | DENV-2 |
| GQ398260 | Indonesia | 1976 | DENV-2 |
| GQ398261 | Indonesia | 1976 | DENV-2 |
| GQ398262 | Indonesia | 1976 | DENV-2 |
| GQ398257 | Indonesia | 1977 | DENV-2 |
| AB189122 | Indonesia | 1998 | DENV-2 |
| AB189123 | Indonesia | 1998 | DENV-2 |
| AB189124 | Indonesia | 1998 | DENV-2 |
| KR604819 | Indonesia | 2007 | DENV-2 |
| KC762665 | Indonesia | 2007 | DENV-2 |
| KC762676 | Indonesia | 2007 | DENV-2 |
| KC762669 | Indonesia | 2007 | DENV-2 |
| KC762658 | Indonesia | 2007 | DENV-2 |
| KC762661 | Indonesia | 2007 | DENV-2 |
| KC762655 | Indonesia | 2007 | DENV-2 |
| KC762662 | Indonesia | 2007 | DENV-2 |
| KC762660 | Indonesia | 2007 | DENV-2 |
| KC762656 | Indonesia | 2007 | DENV-2 |
| KC762666 | Indonesia | 2008 | DENV-2 |
| KC762677 | Indonesia | 2008 | DENV-2 |
| KC762671 | Indonesia | 2008 | DENV-2 |
| KC762663 | Indonesia | 2008 | DENV-2 |
| KC762675 | Indonesia | 2008 | DENV-2 |
| KC762668 | Indonesia | 2008 | DENV-2 |
| KC762673 | Indonesia | 2008 | DENV-2 |
| KC762674 | Indonesia | 2008 | DENV-2 |
| KC762664 | Indonesia | 2008 | DENV-2 |
| KC762672 | Indonesia | 2008 | DENV-2 |
| KC762678 | Indonesia | 2010 | DENV-2 |
| KC762679 | Indonesia | 2010 | DENV-2 |
| KC762680 | Indonesia | 2010 | DENV-2 |
| LC064746 | Indonesia | 2015 | DENV-2 |
| KJ545480 | Malaysia | 2013 | DENV-2 |
| KF041236 | Pakistan | 2008 | DENV-2 |
| KF041237 | Pakistan | 2009 | DENV-2 |
| KF041235 | Pakistan | 2009 | DENV-2 |
| KF360005 | Pakistan | 2010 | DENV-2 |
| KF041234 | Pakistan | 2011 | DENV-2 |
| JX042492 | Pakistan | 2011 | DENV-2 |
| JX042493 | Pakistan | 2011 | DENV-2 |
| JX042496 | Pakistan | 2011 | DENV-2 |
| JX042498 | Pakistan | 2011 | DENV-2 |
| JX042504 | Pakistan | 2011 | DENV-2 |
| JX042508 | Pakistan | 2011 | DENV-2 |
| JX042509 | Pakistan | 2011 | DENV-2 |
| JX042511 | Pakistan | 2011 | DENV-2 |
| JX042514 | Pakistan | 2011 | DENV-2 |
| KM217158 | Pakistan | 2013 | DENV-2 |
| KJ701507 | Pakistan | 2013 | DENV-2 |
| KJ010186 | Pakistan | 2013 | DENV-2 |
| KF744406 | Philippines | 1995 | DENV-2 |
| KF744403 | Philippines | 1995 | DENV-2 |
| KF744404 | Philippines | 1996 | DENV-2 |
| KF744405 | Philippines | 1996 | DENV-2 |
| KF744400 | Philippines | 2000 | DENV-2 |
| KF744401 | Philippines | 2000 | DENV-2 |
| KF744402 | Philippines | 2000 | DENV-2 |
| KF744397 | Philippines | 2001 | DENV-2 |
| AY706007 | Philippines | 2003 | DENV-2 |
| KF744398 | Philippines | 2005 | DENV-2 |
| KF744408 | Philippines | 2005 | DENV-2 |
| JN851123 | Singapore | 2004 | DENV-2 |
| JN851127 | Singapore | 2004 | DENV-2 |
| JN851130 | Singapore | 2005 | DENV-2 |
| JN851131 | Singapore | 2005 | DENV-2 |
| JN851124 | Singapore | 2005 | DENV-2 |
| JN851125 | Singapore | 2005 | DENV-2 |
| JN851126 | Singapore | 2005 | DENV-2 |
| EU081177 | Singapore | 2005 | DENV-2 |
| EU081178 | Singapore | 2005 | DENV-2 |
| EU081179 | Singapore | 2005 | DENV-2 |
| EU081180 | Singapore | 2005 | DENV-2 |
| JN851128 | Singapore | 2006 | DENV-2 |
| JN851129 | Singapore | 2006 | DENV-2 |
| JN851113 | Singapore | 2006 | DENV-2 |
| KM279603 | Singapore | 2007 | DENV-2 |
| KR779782 | Singapore | 2007 | DENV-2 |
| GQ398266 | Singapore | 2007 | DENV-2 |
| GQ398267 | Singapore | 2007 | DENV-2 |
| JN851114 | Singapore | 2007 | DENV-2 |
| JN851115 | Singapore | 2007 | DENV-2 |
| JN851116 | Singapore | 2007 | DENV-2 |
| JN851117 | Singapore | 2007 | DENV-2 |
| JN851120 | Singapore | 2007 | DENV-2 |
| GU370050 | Singapore | 2007 | DENV-2 |
| JN851121 | Singapore | 2008 | DENV-2 |
| JN851122 | Singapore | 2008 | DENV-2 |
| JN851118 | Singapore | 2008 | DENV-2 |
| JN851119 | Singapore | 2008 | DENV-2 |
| GQ398265 | Singapore | 2008 | DENV-2 |
| KM279604 | Singapore | 2008 | DENV-2 |
| KM279602 | Singapore | 2008 | DENV-2 |
| GU370051 | Singapore | 2008 | DENV-2 |
| JF327392 | Singapore | 2009 | DENV-2 |
| KM279605 | Singapore | 2009 | DENV-2 |
| KM279609 | Singapore | 2009 | DENV-2 |
| KM279610 | Singapore | 2010 | DENV-2 |
| KM279606 | Singapore | 2010 | DENV-2 |
| KM279607 | Singapore | 2010 | DENV-2 |
| KM279608 | Singapore | 2010 | DENV-2 |
| KM279583 | Singapore | 2011 | DENV-2 |
| KM279584 | Singapore | 2011 | DENV-2 |
| KM279586 | Singapore | 2011 | DENV-2 |
| KM279513 | Singapore | 2011 | DENV-2 |
| KM279518 | Singapore | 2011 | DENV-2 |
| KM279522 | Singapore | 2011 | DENV-2 |
| KM279524 | Singapore | 2011 | DENV-2 |
| KM279525 | Singapore | 2011 | DENV-2 |
| KM279526 | Singapore | 2011 | DENV-2 |
| KM279527 | Singapore | 2011 | DENV-2 |
| KM279589 | Singapore | 2012 | DENV-2 |
| KM279593 | Singapore | 2012 | DENV-2 |
| KM279594 | Singapore | 2012 | DENV-2 |
| KM279598 | Singapore | 2012 | DENV-2 |
| KM279540 | Singapore | 2012 | DENV-2 |
| KM279550 | Singapore | 2012 | DENV-2 |
| KM279553 | Singapore | 2012 | DENV-2 |
| KM279556 | Singapore | 2012 | DENV-2 |
| KM279563 | Singapore | 2012 | DENV-2 |
| KM279574 | Singapore | 2012 | DENV-2 |
| KM279580 | Singapore | 2013 | DENV-2 |
| KF479405 | Singapore | 2013 | DENV-2 |
| FJ882602 | SriLanka | 1996 | DENV-2 |
| GQ252676 | SriLanka | 2003 | DENV-2 |
| GQ252677 | SriLanka | 2004 | DENV-2 |
| DQ645540 | Taiwan | 2001 | DENV-2 |
| DQ645541 | Taiwan | 2001 | DENV-2 |
| DQ645542 | Taiwan | 2001 | DENV-2 |
| DQ645543 | Taiwan | 2001 | DENV-2 |
| DQ645544 | Taiwan | 2001 | DENV-2 |
| DQ645545 | Taiwan | 2002 | DENV-2 |
| DQ645546 | Taiwan | 2002 | DENV-2 |
| DQ645547 | Taiwan | 2002 | DENV-2 |
| DQ645548 | Taiwan | 2002 | DENV-2 |
| DQ645549 | Taiwan | 2002 | DENV-2 |
| DQ645550 | Taiwan | 2002 | DENV-2 |
| DQ645551 | Taiwan | 2002 | DENV-2 |
| DQ645552 | Taiwan | 2002 | DENV-2 |
| DQ645553 | Taiwan | 2002 | DENV-2 |
| DQ645554 | Taiwan | 2002 | DENV-2 |
| HQ891023 | Taiwan | 2008 | DENV-2 |
| HQ891024 | Taiwan | 2008 | DENV-2 |
| KJ734727 | Taiwan | 2014 | DENV-2 |
| GQ868591 | Thailand | 1964 | DENV-2 |
| GU289914 | Thailand | 1974 | DENV-2 |
| AJ487271 | Thailand | 1974 | DENV-2 |
| DQ181806 | Thailand | 1974 | DENV-2 |
| DQ181805 | Thailand | 1979 | DENV-2 |
| DQ181804 | Thailand | 1984 | DENV-2 |
| DQ181803 | Thailand | 1985 | DENV-2 |
| DQ181802 | Thailand | 1988 | DENV-2 |
| DQ181801 | Thailand | 1990 | DENV-2 |
| EU726767 | Thailand | 1994 | DENV-2 |
| EU687246 | Thailand | 1994 | DENV-2 |
| GQ868542 | Thailand | 1994 | DENV-2 |
| GQ868543 | Thailand | 1995 | DENV-2 |
| DQ181800 | Thailand | 1995 | DENV-2 |
| GQ868544 | Thailand | 1996 | DENV-2 |
| GQ868545 | Thailand | 1996 | DENV-2 |
| FJ906957 | Thailand | 1996 | DENV-2 |
| FJ906958 | Thailand | 1996 | DENV-2 |
| DQ181799 | Thailand | 1998 | DENV-2 |
| DQ181798 | Thailand | 1999 | DENV-2 |
| GU131886 | Thailand | 2001 | DENV-2 |
| FJ744710 | Thailand | 2001 | DENV-2 |
| FJ744714 | Thailand | 2001 | DENV-2 |
| FJ744724 | Thailand | 2001 | DENV-2 |
| FJ639831 | Thailand | 2001 | DENV-2 |
| FJ687435 | Thailand | 2001 | DENV-2 |
| FJ687439 | Thailand | 2001 | DENV-2 |
| FJ687442 | Thailand | 2001 | DENV-2 |
| FJ687445 | Thailand | 2001 | DENV-2 |
| FJ810409 | Thailand | 2001 | DENV-2 |
| FJ898452 | Thailand | 2003 | DENV-2 |
| KJ545481 | Thailand | 2013 | DENV-2 |
| JN819418 | VietNam | 1988 | DENV-2 |
| JX649147 | VietNam | 1995 | DENV-2 |
| JX649148 | VietNam | 1995 | DENV-2 |
| FM210217 | VietNam | 1999 | DENV-2 |
| FM210224 | VietNam | 2001 | DENV-2 |
| FM210225 | VietNam | 2001 | DENV-2 |
| FM210238 | VietNam | 2001 | DENV-2 |
| FM210226 | VietNam | 2002 | DENV-2 |
| FM210227 | VietNam | 2002 | DENV-2 |
| FM210218 | VietNam | 2002 | DENV-2 |
| AB479041 | VietNam | 2002 | DENV-2 |
| AB479042 | VietNam | 2002 | DENV-2 |
| FM210223 | VietNam | 2003 | DENV-2 |
| FM210228 | VietNam | 2003 | DENV-2 |
| FM210230 | VietNam | 2003 | DENV-2 |
| FM210211 | VietNam | 2003 | DENV-2 |
| FM210212 | VietNam | 2003 | DENV-2 |
| FM210203 | VietNam | 2003 | DENV-2 |
| FM210204 | VietNam | 2003 | DENV-2 |
| EU482782 | VietNam | 2003 | DENV-2 |
| EU482783 | VietNam | 2003 | DENV-2 |
| EU482785 | VietNam | 2003 | DENV-2 |
| FM210240 | VietNam | 2004 | DENV-2 |
| FM210242 | VietNam | 2004 | DENV-2 |
| FM210244 | VietNam | 2004 | DENV-2 |
| EU482472 | VietNam | 2004 | DENV-2 |
| FM210215 | VietNam | 2004 | DENV-2 |
| FM210231 | VietNam | 2004 | DENV-2 |
| FM210232 | VietNam | 2004 | DENV-2 |
| FM210236 | VietNam | 2004 | DENV-2 |
| FM210202 | VietNam | 2004 | DENV-2 |
| FM210222 | VietNam | 2004 | DENV-2 |
| FM210235 | VietNam | 2005 | DENV-2 |
| FM210213 | VietNam | 2005 | DENV-2 |
| FM210205 | VietNam | 2005 | DENV-2 |
| FM210206 | VietNam | 2005 | DENV-2 |
| FM210207 | VietNam | 2005 | DENV-2 |
| EU482776 | VietNam | 2005 | DENV-2 |
| EU482777 | VietNam | 2005 | DENV-2 |
| FM210245 | VietNam | 2005 | DENV-2 |
| FM210246 | VietNam | 2005 | DENV-2 |
| EU482473 | VietNam | 2006 | DENV-2 |
| EU482650 | VietNam | 2006 | DENV-2 |
| EU482651 | VietNam | 2006 | DENV-2 |
| EU482653 | VietNam | 2006 | DENV-2 |
| EU482657 | VietNam | 2006 | DENV-2 |
| EU482668 | VietNam | 2006 | DENV-2 |
| EU482676 | VietNam | 2006 | DENV-2 |
| EU482542 | VietNam | 2006 | DENV-2 |
| EU482445 | VietNam | 2006 | DENV-2 |
| EU482447 | VietNam | 2006 | DENV-2 |
| EU677148 | VietNam | 2007 | DENV-2 |
| EU660414 | VietNam | 2007 | DENV-2 |
| EU687249 | VietNam | 2007 | DENV-2 |
| FJ024454 | VietNam | 2007 | DENV-2 |
| EU482704 | VietNam | 2007 | DENV-2 |
| EU482474 | VietNam | 2007 | DENV-2 |
| FJ547067 | VietNam | 2007 | DENV-2 |
| FJ410223 | VietNam | 2007 | DENV-2 |
| FJ461321 | VietNam | 2007 | DENV-2 |
| FJ432726 | VietNam | 2007 | DENV-2 |
| FJ410233 | VietNam | 2008 | DENV-2 |
| FJ461309 | VietNam | 2008 | DENV-2 |
| FJ461311 | VietNam | 2008 | DENV-2 |
| FJ410215 | VietNam | 2008 | DENV-2 |
| FJ410259 | VietNam | 2008 | DENV-2 |
| FJ410237 | VietNam | 2008 | DENV-2 |
| FJ410241 | VietNam | 2008 | DENV-2 |
| KU050695 | Philippines | 1956 | DENV-3 |
| AB609590 | Philippines | 1956 | DENV-3 |
| KM190937 | Philippines | 1964 | DENV-3 |
| JQ922555 | India | 1966 | DENV-3 |
| DQ863638 | Thailand | 1973 | DENV-3 |
| GQ868593 | Thailand | 1973 | DENV-3 |
| GQ199887 | SriLanka | 1983 | DENV-3 |
| GQ199888 | SriLanka | 1983 | DENV-3 |
| GQ199889 | SriLanka | 1983 | DENV-3 |
| KJ737430 | Thailand | 1983 | DENV-3 |
| KF955476 | SriLanka | 1983 | DENV-3 |
| KF955477 | India | 1984 | DENV-3 |
| FJ882574 | SriLanka | 1985 | DENV-3 |
| AY676352 | Thailand | 1987 | DENV-3 |
| AY676353 | Thailand | 1987 | DENV-3 |
| AY858038 | Indonesia | 1988 | DENV-3 |
| JQ411814 | SriLanka | 1989 | DENV-3 |
| FJ882571 | SriLanka | 1989 | DENV-3 |
| FJ882572 | SriLanka | 1989 | DENV-3 |
| KF955474 | SriLanka | 1989 | DENV-3 |
| FJ882573 | SriLanka | 1993 | DENV-3 |
| AY676350 | Thailand | 1993 | DENV-3 |
| AY676351 | Thailand | 1993 | DENV-3 |
| AY876494 | Thailand | 1994 | DENV-3 |
| AY923865 | Thailand | 1994 | DENV-3 |
| KJ737429 | Thailand | 1994 | DENV-3 |
| DQ675519 | Taiwan | 1995 | DENV-3 |
| GQ252674 | SriLanka | 1997 | DENV-3 |
| AY496879 | Philippines | 1997 | DENV-3 |
| DQ675520 | Indonesia | 1998 | DENV-3 |
| DQ675521 | Taiwan | 1998 | DENV-3 |
| DQ675522 | Taiwan | 1998 | DENV-3 |
| DQ675523 | Taiwan | 1998 | DENV-3 |
| DQ675524 | Taiwan | 1998 | DENV-3 |
| DQ675525 | Taiwan | 1998 | DENV-3 |
| DQ675526 | Taiwan | 1998 | DENV-3 |
| DQ675527 | Taiwan | 1998 | DENV-3 |
| DQ675528 | Taiwan | 1998 | DENV-3 |
| DQ675529 | Taiwan | 1998 | DENV-3 |
| DQ675530 | Taiwan | 1998 | DENV-3 |
| AY912454 | Indonesia | 1998 | DENV-3 |
| AB189125 | Indonesia | 1998 | DENV-3 |
| AB189126 | Indonesia | 1998 | DENV-3 |
| AB189127 | Indonesia | 1998 | DENV-3 |
| AB189128 | Indonesia | 1998 | DENV-3 |
| AY676348 | Thailand | 1998 | DENV-3 |
| AY676349 | Thailand | 1998 | DENV-3 |
| AY858039 | Indonesia | 1998 | DENV-3 |
| KF955461 | Cambodia | 1999 | DENV-3 |
| DQ675533 | Taiwan | 1999 | DENV-3 |
| FJ639719 | Cambodia | 2000 | DENV-3 |
| KF955332 | Cambodia | 2000 | DENV-3 |
| GQ868626 | Cambodia | 2001 | DENV-3 |
| KF955462 | Cambodia | 2001 | DENV-3 |
| KF955463 | Cambodia | 2001 | DENV-3 |
| FJ639720 | Cambodia | 2001 | DENV-3 |
| FJ744726 | Thailand | 2001 | DENV-3 |
| FJ744730 | Thailand | 2001 | DENV-3 |
| FJ744732 | Thailand | 2001 | DENV-3 |
| FJ744733 | Thailand | 2001 | DENV-3 |
| FJ744734 | Thailand | 2001 | DENV-3 |
| FJ744736 | Thailand | 2001 | DENV-3 |
| FJ744737 | Thailand | 2001 | DENV-3 |
| FJ744738 | Thailand | 2001 | DENV-3 |
| FJ744740 | Thailand | 2001 | DENV-3 |
| FJ687448 | Thailand | 2001 | DENV-3 |
| FJ639721 | Cambodia | 2002 | DENV-3 |
| FJ639722 | Cambodia | 2002 | DENV-3 |
| AY496871 | Bangladesh | 2002 | DENV-3 |
| AY496873 | Bangladesh | 2002 | DENV-3 |
| AY496874 | Bangladesh | 2002 | DENV-3 |
| AY496877 | Bangladesh | 2002 | DENV-3 |
| GQ868627 | Cambodia | 2002 | DENV-3 |
| GU131906 | Cambodia | 2003 | DENV-3 |
| FJ639723 | Cambodia | 2003 | DENV-3 |
| FJ639724 | Cambodia | 2003 | DENV-3 |
| FJ639725 | Cambodia | 2003 | DENV-3 |
| FJ639726 | Cambodia | 2004 | DENV-3 |
| AY858040 | Indonesia | 2004 | DENV-3 |
| AY858041 | Indonesia | 2004 | DENV-3 |
| AY858042 | Indonesia | 2004 | DENV-3 |
| AY858043 | Indonesia | 2004 | DENV-3 |
| AY858044 | Indonesia | 2004 | DENV-3 |
| AY858045 | Indonesia | 2004 | DENV-3 |
| AY858046 | Indonesia | 2004 | DENV-3 |
| AY858047 | Indonesia | 2004 | DENV-3 |
| AY858048 | Indonesia | 2004 | DENV-3 |
| AY858037 | Indonesia | 2004 | DENV-3 |
| EU081181 | Singapore | 2004 | DENV-3 |
| JQ922556 | India | 2005 | DENV-3 |
| JQ922557 | India | 2005 | DENV-3 |
| GQ868628 | Cambodia | 2005 | DENV-3 |
| GQ868629 | Cambodia | 2005 | DENV-3 |
| GU131904 | Cambodia | 2005 | DENV-3 |
| KF955333 | Cambodia | 2005 | DENV-3 |
| EU081182 | Singapore | 2005 | DENV-3 |
| EU081184 | Singapore | 2005 | DENV-3 |
| EU081195 | Singapore | 2005 | DENV-3 |
| EU081208 | Singapore | 2005 | DENV-3 |
| EU081213 | Singapore | 2005 | DENV-3 |
| EU081216 | Singapore | 2005 | DENV-3 |
| EU081217 | Singapore | 2005 | DENV-3 |
| EU081221 | Singapore | 2005 | DENV-3 |
| EU081222 | Singapore | 2005 | DENV-3 |
| EU081223 | Singapore | 2005 | DENV-3 |
| AB214879 | EastTimor | 2005 | DENV-3 |
| AB214880 | EastTimor | 2005 | DENV-3 |
| AB214881 | EastTimor | 2005 | DENV-3 |
| AB214882 | EastTimor | 2005 | DENV-3 |
| FJ639727 | Cambodia | 2005 | DENV-3 |
| FJ639728 | Cambodia | 2005 | DENV-3 |
| KF041259 | Pakistan | 2006 | DENV-3 |
| KF041256 | Pakistan | 2006 | DENV-3 |
| KF041257 | Pakistan | 2006 | DENV-3 |
| EU660407 | VietNam | 2006 | DENV-3 |
| EU660409 | VietNam | 2006 | DENV-3 |
| EU482452 | VietNam | 2006 | DENV-3 |
| EU482453 | VietNam | 2006 | DENV-3 |
| EU482455 | VietNam | 2006 | DENV-3 |
| EU482456 | VietNam | 2006 | DENV-3 |
| EU482457 | VietNam | 2006 | DENV-3 |
| EU482458 | VietNam | 2006 | DENV-3 |
| EU482459 | VietNam | 2006 | DENV-3 |
| EU482460 | VietNam | 2006 | DENV-3 |
| KF955507 | Cambodia | 2007 | DENV-3 |
| KF955464 | Cambodia | 2007 | DENV-3 |
| KF955457 | VietNam | 2007 | DENV-3 |
| KF955458 | VietNam | 2007 | DENV-3 |
| FJ644564 | India | 2007 | DENV-3 |
| GU131912 | Cambodia | 2007 | DENV-3 |
| GU131913 | Cambodia | 2007 | DENV-3 |
| GU131914 | Cambodia | 2007 | DENV-3 |
| GU131918 | Cambodia | 2007 | DENV-3 |
| HM181935 | Cambodia | 2007 | DENV-3 |
| GU131937 | Cambodia | 2007 | DENV-3 |
| GU131940 | Cambodia | 2007 | DENV-3 |
| GU131943 | Cambodia | 2007 | DENV-3 |
| KF041255 | Pakistan | 2007 | DENV-3 |
| EU482462 | VietNam | 2007 | DENV-3 |
| FJ547061 | VietNam | 2007 | DENV-3 |
| FJ562097 | VietNam | 2007 | DENV-3 |
| FJ562099 | VietNam | 2007 | DENV-3 |
| FJ562102 | VietNam | 2007 | DENV-3 |
| FJ461329 | VietNam | 2007 | DENV-3 |
| FJ432731 | VietNam | 2007 | DENV-3 |
| FJ432743 | VietNam | 2007 | DENV-3 |
| GU370053 | Singapore | 2007 | DENV-3 |
| KC762681 | Indonesia | 2007 | DENV-3 |
| KC762686 | Indonesia | 2007 | DENV-3 |
| KC762684 | Indonesia | 2007 | DENV-3 |
| KC762682 | Indonesia | 2007 | DENV-3 |
| KC762683 | Indonesia | 2007 | DENV-3 |
| KF041254 | Pakistan | 2008 | DENV-3 |
| GQ466079 | India | 2008 | DENV-3 |
| HM631854 | Cambodia | 2008 | DENV-3 |
| GU131946 | Cambodia | 2008 | DENV-3 |
| GU131905 | Cambodia | 2008 | DENV-3 |
| GU131903 | Cambodia | 2008 | DENV-3 |
| KF955459 | VietNam | 2008 | DENV-3 |
| KF955460 | VietNam | 2008 | DENV-3 |
| FJ461322 | VietNam | 2008 | DENV-3 |
| FJ639715 | Cambodia | 2008 | DENV-3 |
| FJ639716 | Cambodia | 2008 | DENV-3 |
| FJ461334 | VietNam | 2008 | DENV-3 |
| FJ461337 | VietNam | 2008 | DENV-3 |
| FJ461338 | VietNam | 2008 | DENV-3 |
| FJ562103 | VietNam | 2008 | DENV-3 |
| FJ547066 | VietNam | 2008 | DENV-3 |
| KC762685 | Indonesia | 2008 | DENV-3 |
| KC762687 | Indonesia | 2008 | DENV-3 |
| KC762689 | Indonesia | 2008 | DENV-3 |
| KC762691 | Indonesia | 2008 | DENV-3 |
| KC762688 | Indonesia | 2008 | DENV-3 |
| KC762690 | Indonesia | 2008 | DENV-3 |
| KF041258 | Pakistan | 2009 | DENV-3 |
| JN380809 | Singapore | 2009 | DENV-3 |
| JN380810 | Singapore | 2009 | DENV-3 |
| GU189648 | China | 2009 | DENV-3 |
| GU370052 | Singapore | 2009 | DENV-3 |
| JN662391 | China | 2009 | DENV-3 |
| GU363549 | China | 2009 | DENV-3 |
| JF504679 | China | 2009 | DENV-3 |
| HG316483 | Thailand | 2010 | DENV-3 |
| HG316484 | Thailand | 2010 | DENV-3 |
| KC762692 | Indonesia | 2010 | DENV-3 |
| KC762693 | Indonesia | 2010 | DENV-3 |
| KF543274 | Cambodia | 2011 | DENV-3 |
| KC261634 | China | 2012 | DENV-3 |
| KJ622191 | China | 2013 | DENV-3 |
| KJ622192 | China | 2013 | DENV-3 |
| KJ622193 | China | 2013 | DENV-3 |
| KJ622194 | China | 2013 | DENV-3 |
| KJ622195 | China | 2013 | DENV-3 |
| KJ622198 | China | 2013 | DENV-3 |
| KF824902 | China | 2013 | DENV-3 |
| KF824903 | China | 2013 | DENV-3 |
| KF954945 | China | 2013 | DENV-3 |
| KF954949 | China | 2013 | DENV-3 |
| KU216208 | India | 2013 | DENV-3 |
| KU216209 | India | 2013 | DENV-3 |
