# Supplementary material for: Increasing airline travel may facilitate co-circulation of multiple dengue virus serotypes in Asia
Source: PLoS Negl Trop Dis. 2017 Aug 3;11(8):e0005694. doi: 10.1371/journal.pntd.0005694 (PMC5542384; doi:10.1371/journal.pntd.0005694)
Supplement: S2 Table — (DOCX) [file pntd.0005694.s008.docx]

**S2 Table** Strongly supported DENV migrations estimated from E gene segment

| Area-A | Area-B | Indicator | BF* | Serotype |
| --- | --- | --- | --- | --- |
| Laos | Thailand | 1 | >1000 | DENV-1 |
| Cambodia | VietNam | 1 | >1000 | DENV-1 |
| China | Singapore | 1 | >1000 | DENV-1 |
| Myanmar | Thailand | 0.99 | 784 | DENV-1 |
| China | Thailand | 0.97 | 166 | DENV-1 |
| India | Thailand | 0.97 | 161 | DENV-1 |
| China | Japan | 0.93 | 79 | DENV-1 |
| China | Indonesia | 0.9 | 53 | DENV-1 |
| Cambodia | Thailand | 0.89 | 47 | DENV-1 |
| Indonesia | Singapore | 1 | >1000 | DENV-2 |
| Cambodia | Thailand | 1 | >1000 | DENV-2 |
| China | Indonesia | 1 | >1000 | DENV-2 |
| Philippines | China Taiwan | 0.98 | 276 | DENV-2 |
| Brunei | Indonesia | 0.95 | 109 | DENV-2 |
| India | SriLanka | 0.94 | 87 | DENV-2 |
| China | Thailand | 0.94 | 87 | DENV-2 |
| India | SriLanka | 0.99 | 851 | DENV-3 |
| Singapore | China Taiwan | 0.95 | 104 | DENV-3 |
| Saudi Arabia | Singapore | 0.94 | 84 | DENV-3 |
| Cambodia | VietNam | 0.91 | 59 | DENV-3 |
| Cambodia | Thailand | 0.91 | 55 | DENV-3 |
| India | Pakistan | 0.9 | 51 | DENV-3 |
| China | Thailand | 0.89 | 48 | DENV-3 |
| China Taiwan | Thailand | 0.87 | 39 | DENV-3 |

Only statistically supported migrations with indicator values >0.50 and BF >30 are shown.
